# Human remains from Arma di Nasino (Liguria) provide novel insights into the paleoecology of early Holocene foragers in northwestern Italy

**DOI:** 10.1038/s41598-023-40438-5

**Published:** 2023-09-29

**Authors:** Vitale S. Sparacello, Gwenaëlle Goude, Alessandra Varalli, Irene Dori, Claudine Gravel-Miguel, Julien Riel-Salvatore, Sanne W. L. Palstra, Jacopo Moggi-Cecchi, Fabio Negrino, Elisabetta Starnini

**Affiliations:** 1 Dipartimento di Scienze della Vita e dell’Ambiente, Sezione di Neuroscienze e Antropologia, Università degli Studi di Cagliari, Cagliari, Italy; 2 CNRS, Aix Marseille Univ, Minist Culture, LAMPEA, UMR 7269, 5 rue du Château de l’Horloge, 13097 Aix-en-Provence, France; 3 CASEs Research Group, Department of Humanities, Universitat Pompeu Fabra, Barcelona, Spain; 4 Dipartimento di Biologia, Università degli Studi di Firenze, Florence, Italy; 5 Département d’Anthropologie, Université de Montréal, Montréal, QC Canada; 6 Center for Applied Fire and Ecosystem Science, New Mexico Consortium, Los Alamos, NM USA; 7 Centre for Isotope Research, ESRIG, University of Groningen, Groningen, The Netherlands; 8 DAFIST - Dipartimento di Antichità, Filosofia, Storia, Università degli Studi di Genova, Genoa, Italy; 9 Dipartimento di Civiltà e Forme Del Sapere, Università di Pisa, Pisa, Italy

**Keywords:** Palaeoecology, Biochemistry, Skeleton

## Abstract

We report the discovery and analysis of new Mesolithic human remains—dated to ca. 10,200–9000 cal. BP—from Arma di Nasino in Liguria, northwestern Italy, an area rich in Upper Paleolithic and Neolithic attestations, but for which little information on Early Holocene occupation was available. The multi-proxy isotopic profile of the two individuals reveals that—despite the proximity of the site to the Mediterranean seashore and the use of shellfish as decorative elements in burials—the ecology of these foragers was based on the exploitation of high-altitude resources, presumably in the nearby western Alps. This constitutes the first direct evidence in northwestern Italy of a significant ecological shift towards higher altitudes following deglaciation, especially when compared to isotopic data of the Late Pleistocene hunter-gatherers from the nearby site of Arene Candide Cave, who exploited terrestrial resources nearer to the coast and at lower altitudes. While the biochemistry of Nasino’s skeletal assemblage revealed new details on Early Holocene lifeways in the area, the osteobiography of one individual offers glimpses into the life experience of a specific female forager, depicting a scenario of early skeletal trauma, developmental disturbances, long-term impairments, and resilience amongst the last European hunter-gatherers.

## Introduction

Between the beginning of the Holocene (11,700 cal. BP^1^) and the diffusion of the Neolithic in Europe, Mesolithic foragers adapted to the rapidly warming climate by modifying the subsistence patterns that had characterized the preceding Upper Paleolithic, or by adopting new ones altogether^2,3^. As the land was freed from ice by the warming climate, new high-altitude hunting grounds began to be exploited in mountainous areas such as the Alps, while in coastal areas of the Atlantic façade and in the southern Baltic region, the exploitation of coastal resources—primarily shellfish—became important^3^. However, our knowledge about the paleoecology of Early Mesolithic foragers (ca. 1100–8500 cal. BP) is often limited and biased by the facts that most sites are surface artifact scatters^2^, and that most of the coastal sites occupied in the earlier phases are now under water^3,4^. In this context, human remains and burials can provide rare glimpses of direct information on several aspects of human biocultural adaptations that most sites cannot, such as diet^5,6^, mobility and activity patterns^7–9^, and social complexity^3,10^.

Unfortunately, the distribution of funerary sites is extremely uneven across Europe: although cemeteries are present since the Early Mesolithic^11^, they have mostly been found in regions where the exploitation of predictable marine and riverine resources favored demographic growth, reduced mobility, and larger settlements^4^, such as northwestern Europe, the lower Danube, and the Dnieper basin^12–14^. In contrast, burials are exceedingly rare in areas such as the Italian Alps and Apennines, where subsistence appears to have focused mainly on the exploitation of terrestrial resources^15^, with small groups alternating seasonally between high pastures and lowlands^16,17^. In fact, in all of northern Italy, only two burials are currently securely dated to the Early Mesolithic (Vatte di Zambana, and Arma Veirana, see below; Mondeval de Sora has been dated to the Late Mesolithic, and recent studies suggest that Mezzocorona-Borgonuovo may belong to the same phase)^6,18–21^. The present study thus adds critically important new data to the meager record of Early Mesolithic burials from Northern Italy by presenting new chronological, biochemical, and biomechanical data from two buried individuals from Liguria now confirmed to also date to this period.

Liguria is a mountainous coastal region in northwestern Italy and one the of the most important regions in Europe for our understanding of terminal Pleistocene human paleobiology, with numerous Upper Paleolithic burials unearthed from several renowned sites such as the Balzi Rossi and Arene Candide caves^22–25^. The Ligurian region is also paramount for the study of the Neolithization of the western Mediterranean, with several caves in the Finalese area (e.g., Arene Candide Cave, Arma Pollera, Arma dell’Aquila) yielding dozens of burials dated to its earliest colonization by agropastoralists^26,27^. Yet, very little is known about the Mesolithic in this pivotal region because archaeological assemblages securely dated to this period are extremely rare: a few artifacts attributable to the Early Mesolithic (the so-called Sauveterrian culture) have been found in surface scatters, without secure chronostratigraphic contexts^28,29^. Moreover, some charcoal samples from caves in eastern and western Liguria have yielded lower Holocene dates (Greenlandian stage 11,700–8236 cal. BP^30^) that could indicate Mesolithic frequentation. However, these dates are incoherent with the associated lithic assemblages, which are attributed to the Late Epigravettian (final Pleistocene), suggesting post-depositional disturbances of inaccurate stratigraphic excavations^29,31^.

The recent discovery of a neonate burial at the site of Arma Veirana, in the Neva valley of the Ligurian Prealps (province of Savona), about 14 km from the current seashore, was the first Mesolithic human unearthed in the region^20^. Dated to 10,211–9914 cal BP (95.4% probability), this important discovery demonstrates that at least some of the numerous caves of western Liguria were used as funerary sites by Early Mesolithic people. The presence of *Columbella rustica* shell beads in the burial attests of a possible connection with the nearby coast. However, being an infant, this burial provides only little information on the subsistence of Mesolithic foragers in the area. As such, burials of Mesolithic adults are still necessary to reconstruct the lifeways of these groups from their skeletons.

In this paper, we report and analyze newly identified Mesolithic human remains, including a burial, from Arma di Nasino (Pennavaira Valley, province of Savona) located in western Liguria, about 4 km west of Arma Veirana. Rather than originating from new excavations, this discovery derives from a re-assessment of previously excavated collections: the remains were previously attributed to the Neolithic (see below), but an early Holocene date was obtained in the context of a recent large-scale campaign of radiocarbon dating on the Ligurian prehistoric skeletal collections^25–27^. Given the importance of these remains for our understanding of the Early Mesolithic occupation of Liguria, we performed further radiocarbon determinations, analyzed the isotopic composition, and contextualized the osteobiography of the Nasino individuals, including activity patterns and pathological conditions, within our current understanding of human biocultural adaptations at that time.

## Results

### The site and the location of skeletal remains

**Figure 1:**
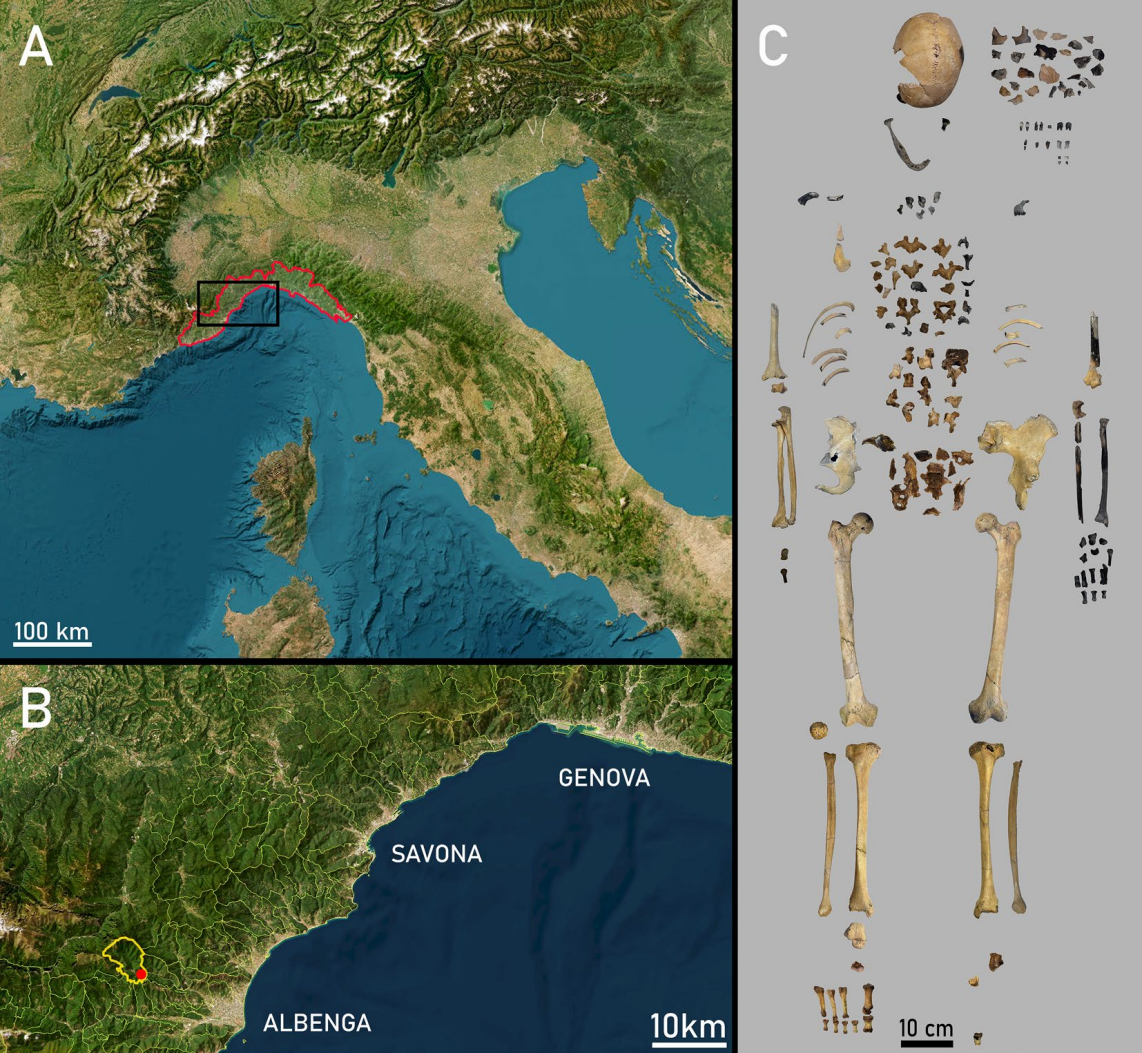
The geographic location and the human remains of Nasino 2. (**A**) Map of the geographic location of Liguria in northwestern Italy (Modified from ESRI World Imagery CC BY 4.0; https://srvcarto.regione.liguria.it). (**B**) Magnification of the area within the black rectangle in (**A**), showing the location of the municipality of Nasino and the main Ligurian cities nearby. (**C**) Photographic collage of the skeletal elements belonging to Nasino 2. Pictures taken by VSS.

Arma di Nasino, or Nasino Cave (“Arma” is a synonym for “cave” used as shelter or refuge in the local dialect) was a wide and shallow shelter that opened on the eastern flank of the Pennavaire valley, 246 m a.s.l. and ca. 14.5 km inland from the coastal town of Albenga (province of Savona; Fig. 1). Unfortunately, the shelter has now been destroyed by quarrying activities. Similarly to the nearby Finalese area, several shelters and caves in this and neighboring valleys have yielded archaeological assemblages ranging from the Late Pleistocene to historic times^25–27^. At Arma di Nasino, the in-situ stratigraphy spanned the Late Epigravettian to the end of the Bronze Age, while the disturbed upper layers contained scarce roman and modern artifacts^32^. Excavations were conducted by Leale Anfossi between 1961 and 1974^33^, and yielded two burials, one partial skeleton, and a small assemblage of scattered human remains^32^. The first burial (Nasino 1) was found in 1967, crouched on its left side against the eastern wall of the cave, and was attributed to the Neolithic^34^. A later direct date confirmed it dated to the end of the fifth millennium BCE, compatible with a Chassean chrono-cultural attribution^27^. The skeletal materials that are the object of this study were originally recovered in 1968, and originally also attributed to the Neolithic based on the depth at which they were found^32,35^.

The second burial (Nasino 2) was found close to the mouth of the cave and consists of a supine individual with extended limbs, deposited on what was described as a “bed” of stones and surrounded by other rocks^32^. About a hundred shells of pierced *Columbella rustica* were arranged around the individual, especially in the pelvic area^35^. A few bones of a third individual (Nasino 3), not in anatomical connection, were found in the northwestern corner of the cave against the wall (Supplementary Information 1).

### Radiocarbon dating

Three AMS radiocarbon dates were performed on three different fragments of bone from Nasino 2 and two from Nasino 3 (Table 1). All bone samples yielded an adequate amount of collagen (> 1% of the sample weight) respecting the isotopic criteria for a reliable date (C > 30%, N > 10%, C/N between 2.9 and 3.6). All dates confirm an Early Holocene chronological attribution of the remains. For Nasino 2 we obtained two overlapping dates placing the individual at 9399–9033 cal. BP (95.4% probability; dates combined using OxCal 4.4.4); considering a 88.1% probability, date would fall at 9306–9121 cal. BP. The third date obtained for Nasino 2, placing this individual at 9548–9486 cal. BP (95.4% probability), does not overlap and cannot be combined with the previous ones (failed X^2^-Test at 5%). Although the fragment of cortical bone used for this date was already detached when sampled, it appears to belong to the reconstructed portion of the right femur of Nasino 2 (Fig. 1) and not to another individual. Slight discrepancies when performing multiple dates on the same individual have been observed in previous studies; more research is necessary, but it has been hypothesized that they may be the result of contamination that does not significantly alter the isotopic values^21,25–27^. Conversely, the two dates obtained for Nasino 3 are highly consistent, and their combination places the individual at 10,170–9900 cal. BP (95.4% probability; dates combined using OxCal 4.4.4).

**Table 1:** Radiocarbon dating and isotopic composition of the Nasino individuals analyzed in this paper. Further details are available in SI 1.

Lab code	Individual	Nitrogen content %	Carbon content %	C/N	δ^15^N AIR	δ^13^CV PDB	Sulfur content %	δ^34^SV CDT	C/S	N/S	^14^C age (yr BP)	Cal BP 95.4%
GrM-13521	Nasino 2	11.99	33.13	3.2	7.40	− 19.98	0.16	4.9	547.6	170.0	8236 ± 30	9400–9030
GrM-15944	Nasino 2	15	43.4	3.3	7.55	− 19.89					8555 ± 30	9548–9486
GrM-21897	Nasino 2	14.9	40.8	3.2	7.35	− 19.85					8245 ± 35	9404–9031
GrM-13522	Nasino 3	13.70	37.54	3.2	7.18	− 20.39	0.17	3.7	601.6	188.3	8875 ± 30	10,175–9799
GrM-21898	Nasino 3	14.8	40.5	3.20	6.98	− 20.58					8910 ± 35	10,184–9905

### The skeletal material and biological profile

Both Nasino 2 and 3 consist of partial and fragmentary skeletons, whose composition is summarized in Table 2. Nasino 2, represented in Fig. 1, is the most complete individual of the two, all the regions of the skeleton being preserved; unfortunately, the left upper thoracic and cranial regions show heat damage and blackening due to the later emplacement of a hearth in the layers above the burial^32^.

**Table 2:** – Osteological composition of the Nasino individuals analyzed in this paper.

Individual	Summary osteological composition
Nasino 2	Cranium and mandible: frontal (d); parietal (f/f); occipital (f); temporal (ff/ff); zygomatic (i/d); fragments (> 10ff); mandible (f, 1ff). Teeth: UP1, ULM1, LRC, LLC, LRP, LLM2, LLM3, LRM3, fragments (4ff). Infracranium: clavicle (-/ff); scapula (ff/ff); humerus (f/f); radius (i/i); ulna (f/i); carpals (4i,1d/1i); MC (3f); hand phalanges (3i,1d,3f); cervical vert. (6ff); thoracic vert. (1d, 25ff); lumbar vert. (1d,10ff); sacrum (12ff); os coxae (f/f); femur (i/d); patella; (-/f); tibia (i/i); fibula (d/i); tarsals (-/1i,1d,1ff); MT (1ff/2i,2d); foot phalanges (1f/2d,3i); ribs (> 10ff); fragments (> 10ff)
Nasino 3	Cranial fragments (ff); cervical vert. (ff); scapula (-/ff); humerus (ff); radius (-/ff); os coxae (ff/ff); talus (2ff./f); MTV (-/f)

Side of the element is indicated based on the position with respect to the slash, i.e. (left/right) when appropriate; preservation is indicated by: i (intact or minimally damaged); d (damaged); f (fragment); ff (small fragment).

Biological sex can be determined to have been female for Nasino 2, while no diagnostic information is available for Nasino 3. Both individuals were fully adult at the time of death. On Nasino 2, visible cranial sutures are open, suggesting a young age, but the root apex in the lower third molar is closed, indicating an age above 23.5 years^36^.

### Isotopic composition: diet and mobility

**Figure 2:**
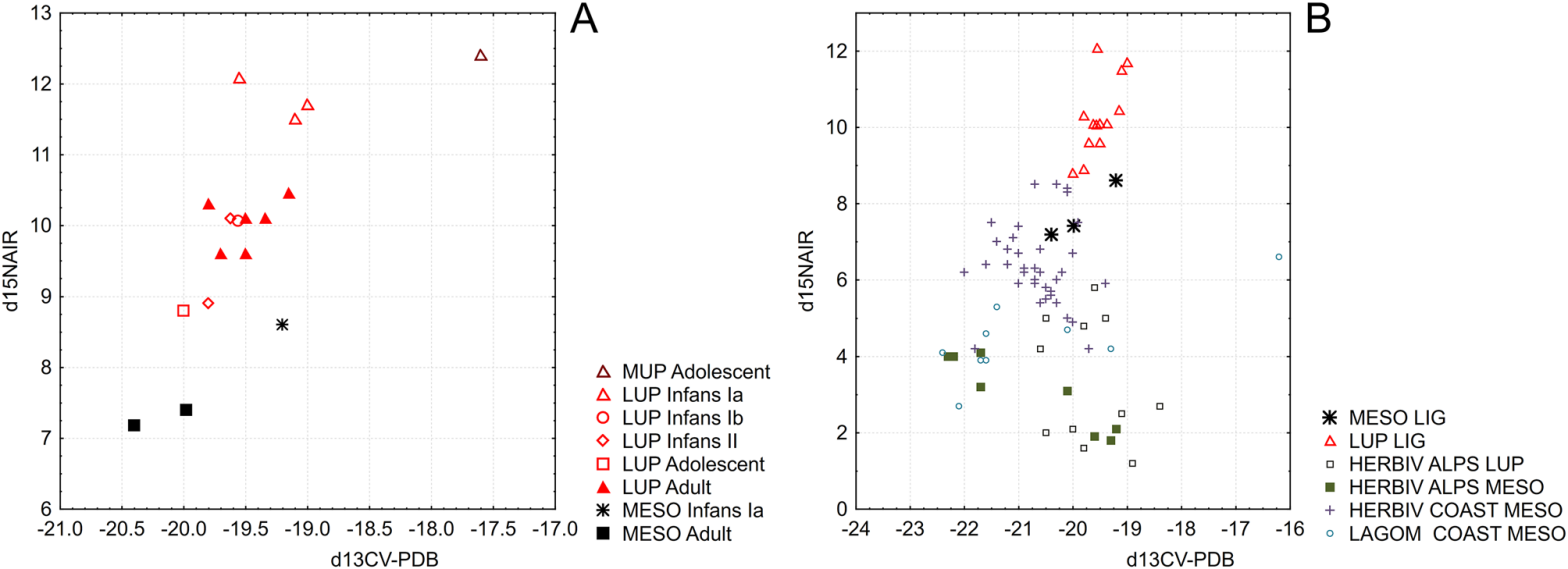
The carbon and nitrogen isotopic values of the Mesolithic and Upper Paleolithic foragers from western Liguria analyzed in this study. (**A**) Scatterplot of the δ^15^N on δ^13^C values, categorized by period and age; MUP, Middle Upper Paleolithic; LUP, Late Upper Paleolithic; MESO, Mesolithic. Age categories; Infans Ia, birth to 2 years; Infans Ib, 2–7 years; Infans II, 7–14 years; Adolescent, until closure of spheno-occipital synchondrosis. (**B**) Scatterplot of the δ^15^N on δ^13^C values for the Ligurian Mesolithic and Late Upper Paleolithic foragers, and for Mesolithic and Late Upper Paleolithic comparative faunal remains (HERBIV: includes ungulates and other herbivores; LAGOM: includes lagomorphs; details in SI 2) from coastal (COAST) and Alpine (ALPS) regions.

The two Early Mesolithic adults from Nasino have similar bone isotopic composition from -20.0 to -20.4‰ for δ^13^C, 7.4 to 7.2‰ for δ^15^N and 3.7 to 4.9‰ for δ^34^S. Unfortunately, due to the excavation methods used at Arma di Nasino, it was not possible to determine a definitely coeval animal baseline to contextualize these results. We therefore investigated the diet of the two adult Mesolithic individuals by comparing them with earlier Upper Paleolithic foragers that lived in the same area: the Late Epigravettian individuals (n = 13) from the Arene Candide Cave necropolis, dated between 12,800–12,500 and 12,100–11,800 cal. BP^25^, analyzed for this study, and the published data for the Gravettian “Il Principe” (“The Prince”) from Arene Candide Cave, dated to ca. 27,900–27,300 cal. BP^37^ (Fig. 2A; raw data in Supplementary Information 2). The carbon isotopic composition of the Late Epigravettian individuals range from − 20 to − 19‰, while δ^15^N data ranges from 8.8 to 10.5‰ in adults, and from 11.5 to 12.1‰ in three infants below 2 years of age; the latter’s collagen composition suggests both the incorporation of maternal milk and growth effect on bone protein turnover (Fig. 2A; review in Herrscher et al.^38^. The trophic position of Late Epigravettian individuals from Liguria is consistent with what is already recorded in the western Mediterranean, i.e., a significant terrestrial animal protein intake but an absent or insignificant role of marine resources in the dietary protein^39^. According to Pettitt et al.^37^, the carbon and nitrogen isotopic composition of the Gravettian individual indicates that approximately 20–25% of dietary protein intake was from marine protein.

The δ^13^C values for the adult Mesolithic individuals from Nasino suggest a highly terrestrial diet^40^, while the δ^15^N values are markedly lower than the Late Epigravettian ones, highlighting the difference in diet or environmental conditions between Late Pleistocene and Early Holocene foragers in the region. One possibility is that a significant portion of dietary protein consumed by Ligurian Mesolithic foragers derived from plants. Alternatively, assuming a subsistence mainly based on exploiting terrestrial animals (cf.^6,41^), the Nasino individuals could have hunted in a different area, in which δ^15^N levels were significantly lower throughout the trophic chain, driven by environmental factors affecting soils, such as precipitation, temperature, and altitude^42,43^. Similar differences were recorded for roe deer living in forest environments, due to acidic pH leading to low nitrogen isotopic composition in soils^44^.

To investigate this possibility, we compared the results obtained for Nasino with published data for wild herbivores from Mesolithic and Upper Paleolithic sites near the Mediterranean Coast (Sicily, Corsica, and Croatia) and from the Italian Alps (Veneto and Trentino regions; raw data in Supplementary Information 2). Figure 2B shows how herbivores from the Italian Alps can present δ^15^N levels which can represent a trophic step below (ca. − 3‰) the Nasino humans. Values of δ^15^N below 2.5‰ are shown by all *Capra ibex* (Mesolithic and Upper Paleolithic) in the comparative sample, and by some Upper Paleolithic *Cervus elaphus*.

**Figure 3:**
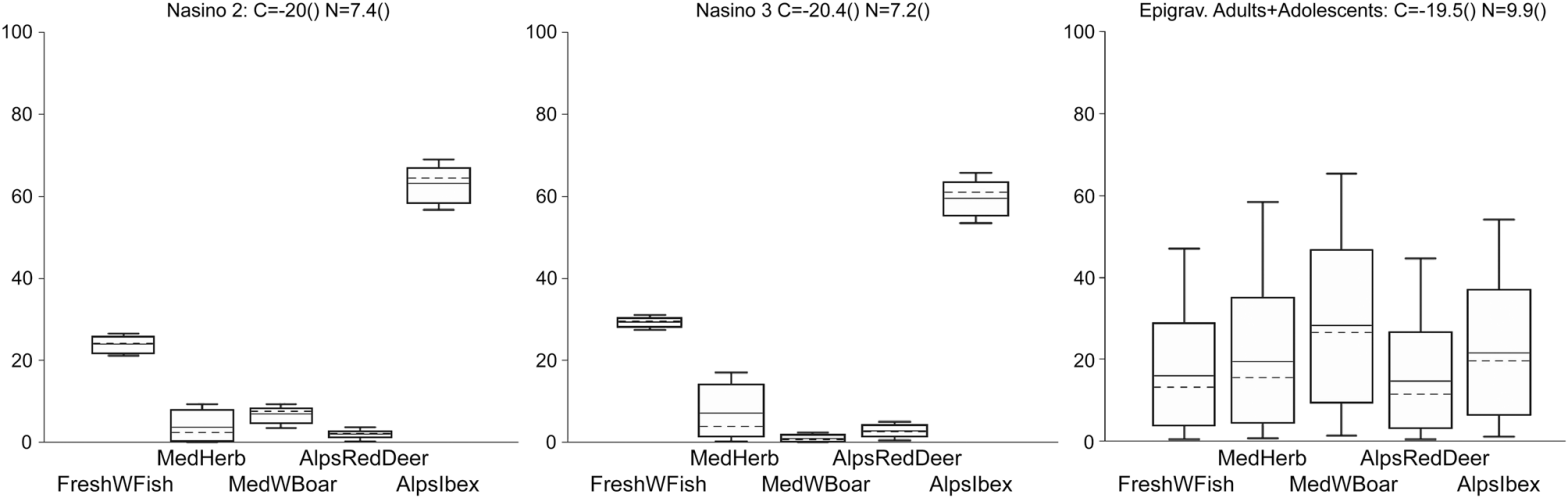
Estimate percentage of the provenience for the protein consumed by Nasino individuals and Ligurian Late Epigravettian adults/adolescents using a Bayesian model (FRUITS) based on five food resources, selected according to the availability of data and relevancy for the chronology and location of the sites (details in SI 1). Boxes represent a 68% credible interval (corresponding to the 16th and 84th percentiles) while the whiskers represent a 95% credible interval (corresponding to the 2.5th and 97.5th percentiles). The horizontal continuous line represents the estimated mean while the horizontal dashed line represents the estimated median (50th percentile).

In order to further investigate this issue, we employed a Bayesian model^45^ (FRUITS; Food Reconstruction Using Isotopic Transferred Signals; https://sourceforge.net/projects/fruits) to estimate the provenience of protein consumed between Ligurian Mesolithic and Late Epigravettian foragers. FRUITS compares a mixed target to several isotopically unique sources, and calculates the most probable relative contribution of each source to the diet of the target. Protein sources considered in the model were freshwater fish, wild herbivores dwelling near the Mediterranean coast (red deer, aurochs, and boar), and wild herbivores dwelling on the Alps (red deer and ibex). Isotopic values for targeted animal resources, targets and information about the model data implemented are provided in Supplementary Information 2. For both Nasino individuals, the model suggests the consumption of freshwater fish and ibex, an animal that tends to dwell above the tree line. This is in keeping with what has been identified at other inland and high-altitude Italian Mesolithic sites^6^. Results for the Late Epigravettian foragers from Arene Candide are compatible with a more diverse diet including animals living in forested environments (Fig. 3).

In Fig. 2, the carbon and nitrogen values obtained for the perinatal individual from Arma Veirana^20^; (δ^13^C: − 19.2‰, δ^15^N: 8.6‰; asterisk in Fig. 2A), coeval to Nasino, are reported. If we consider Nasino values as representative of Ligurian Mesolithic adults living in similar environmental conditions, the data for the 40–50 days-old infant from Arma Veirana would be compatible with the initial effect of breastfeeding^38^.

**Figure 4:**
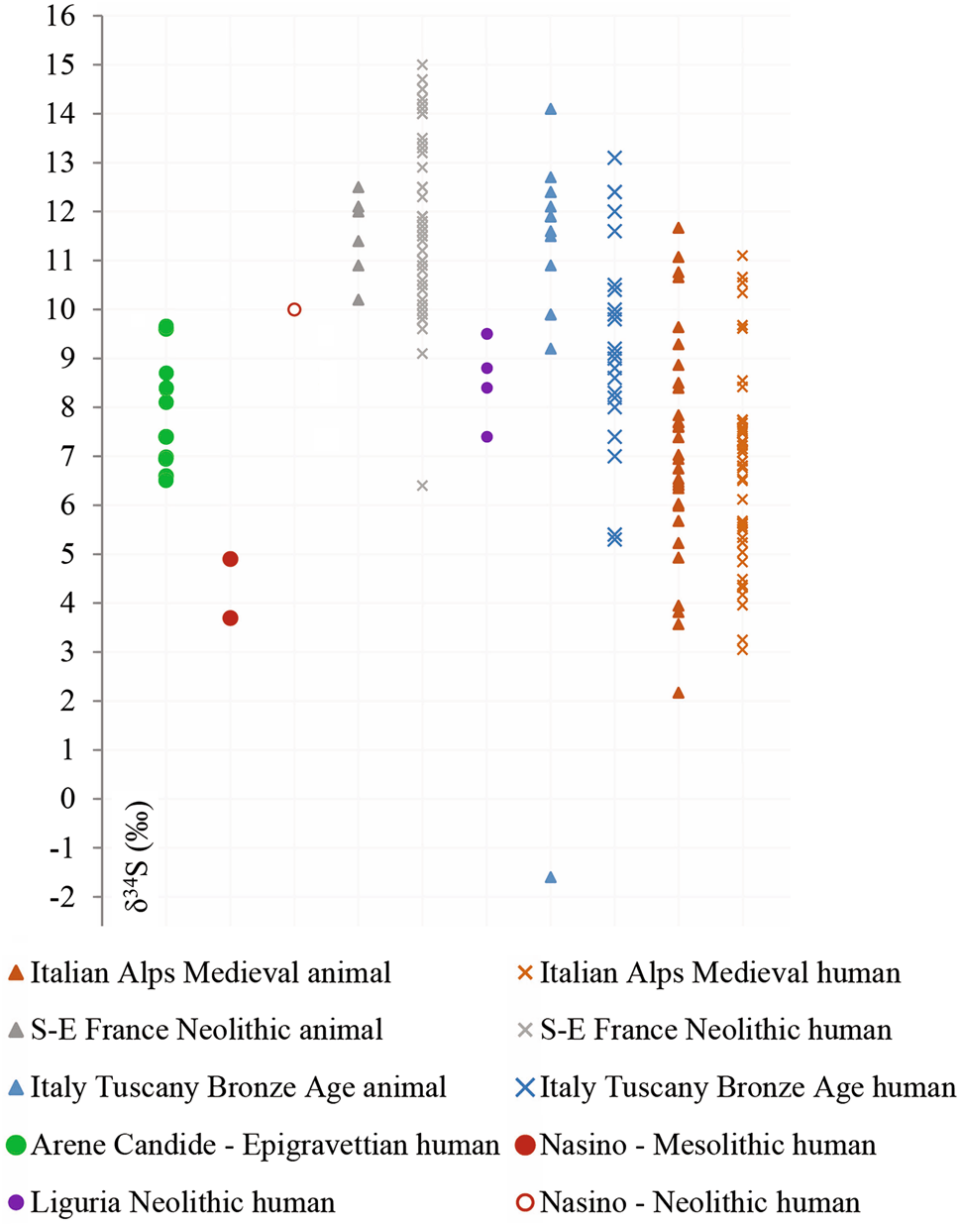
Sulfur (δ^34^S) isotope composition of prehistoric humans from Liguria (Late Upper Paleolithic foragers from Arene Candide, Mesolithic foragers from Nasino; Neolithic agropastoralists from Nasino, Arene Candide, and Arma dell’Aquila) compared with published human and faunal data from archaeological sites located in coastal and Alpine environments in the northern Italian peninsula and southeastern France.

Combined with carbon and nitrogen, sulfur isotope ratios (δ^34^S) integrate information on the food consumed and on the role of marine vs. terrestrial foods. Furthermore, this stable isotope provides environmental information, allowing for a better evaluation of possible differences in ecosystems exploited by Mesolithic foragers (review in Nehlich^46^). Indeed, sulfur isotopic composition decreases in plants and in animal tissues^47^ with increasing distance from the sea due to a lower contribution of ^34^S-enriched marine aerosols^46^. This has been recently explored in an investigation of Neolithic and metal ages livestock mobility in Croatia, where high δ^34^S values (up to 16.6‰) are recorded in herbivores located close to the seashore, while same species provide very low data (up to − 0.8‰) when raised inland^48^. However, to our knowledge, there is no available sulfur data for other prehistoric hunter-gatherers in the western Mediterranean. More recent periods have been investigated and can provide a sub-local baseline^49–55^. Figure 4 shows that the Nasino humans have significantly lower δ^34^S values than Late Epigravettian and Neolithic humans who dwelled in the same region, including the late Neolithic individual from Arma di Nasino. Values similar to the ones shown by the Mesolithic humans from Nasino can be found in humans and animals from the Western^55^ and Eastern Italian Alps (Fig. 4), supporting the interpretation of a terrestrial diet mainly based on hunting inland and at high altitudes for Ligurian Mesolithic foragers.

### Paleopathology

**Figure 5:**
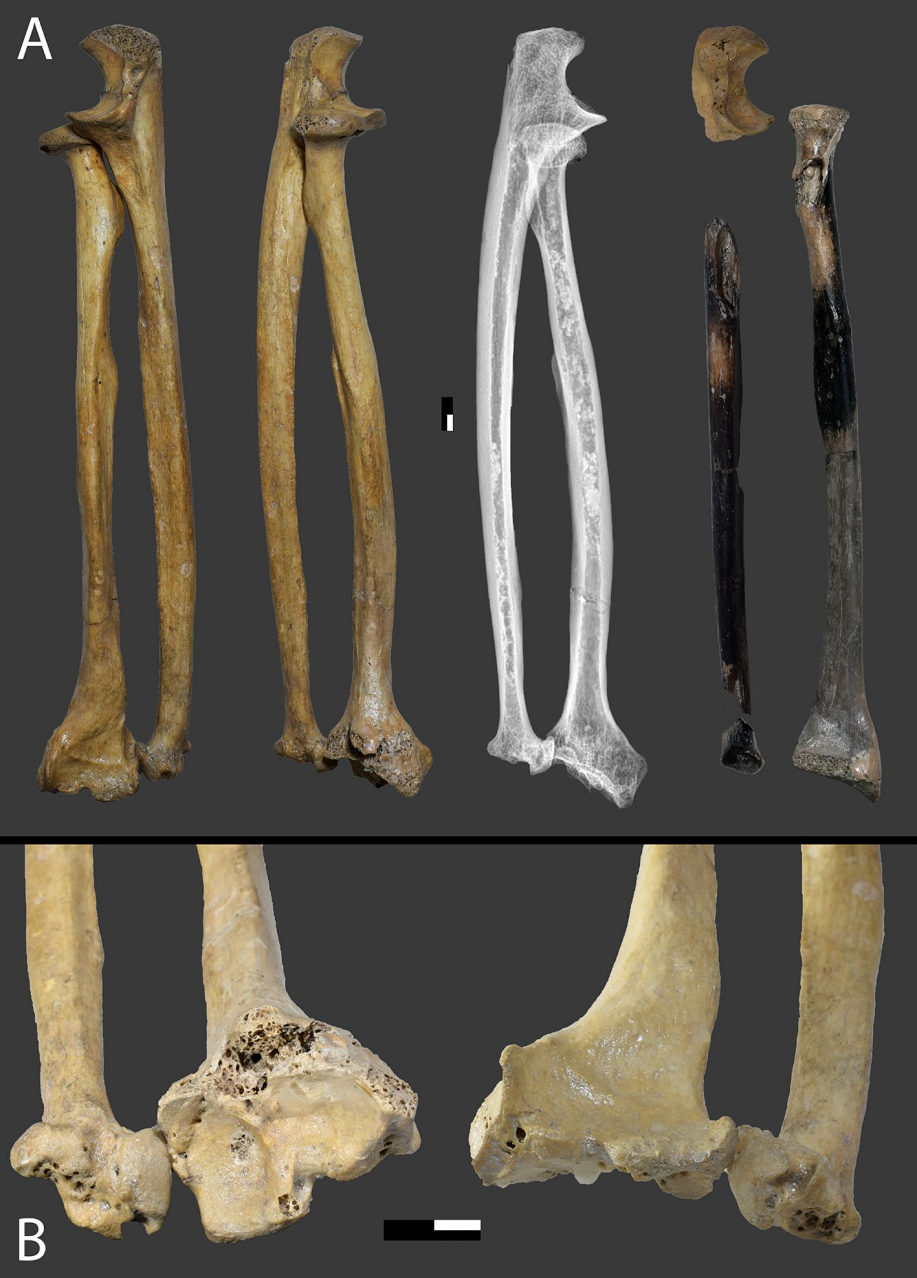
Morphological changes due to trauma in Nasino 2 forearm. (**A**) The right forearm of Nasino 2 in anterior radial, posterior radial view, and radiographic image of the articulated forearm, compared to the contralateral elements (frontal view). (**B**) Detail of the alterations in the radial and ulnar distal epiphyses. Scale: black bar indicates centimeters, white/black bar indicates 5 mm.

Nasino 2 presents a healed and malunited fracture in the right distal forearm, involving both the radius and the ulna (Fig. 5). Distally, the radius shows gross deformity of the metaphysis, epiphysis, and articular surface, with irregular and enlarged lunar and scaphoid facets, accentuated inclination, dorsal tilting and slight shortening leading to positive ulnar variance. Given the degree of curvature of the metaphysis, further accentuated in the dorsal epiphysis, the radial trauma could be described as a compound traumatic bone deformity and a healed Colles fracture^56–58^. The deformities can be best appreciated in the surface 3D model (available at www.morphosource.org). The distal ulna presents a heavily displaced Type I Salter–Harris fracture, with loss of the styloid process and a deformed articular portion that fused in the radial portion of the epiphyseal plate (Fig. 5). Given the presence of a Salter–Harris fracture, the injury must have occurred when the ulnar growth plate was still unfused, i.e. before 15–19 years of age^59^. However, the minimal loss in longitudinal length of the ulna when compared to the contralateral side suggests that the bones were fractured shortly before fusion. Indeed, displaced ulnar Type I Salter–Harris fractures frequently lead to physeal growth arrest^60^.

This type of fracture usually results from injuries suffered by falling on the outstretched hand. Ligamentous disruption of the radioulnar joint and triangular fibrocartilage complex injuries are common, especially when secondary ulnar Salter–Harris fractures and styloid nonunion are present, and neuropathies can develop^58^. Malunited fractures can lead to permanent impairments, especially when they occur in later adolescence^61^, in particular loss of pronation and supination ranges of motion^62^.

### Body proportions, long bone structural analysis, and activity patterns

Considering the femoral head superoinferior diameter as a proxy for body mass, and femoral maximum length as a proxy for stature, Nasino 2 shows body proportions that are more compatible with the Italian Mesolithic sample than with the Ligurian Neolithic females, or with Pleistocene humans (detailed analysis in Supplementary Information 3). Her estimated body mass is ca. 62 kg^63^ while the stature was ca. 152.5 cm (applying the regression equations in Formicola and Franceschi^64^).

**Figure 6:**
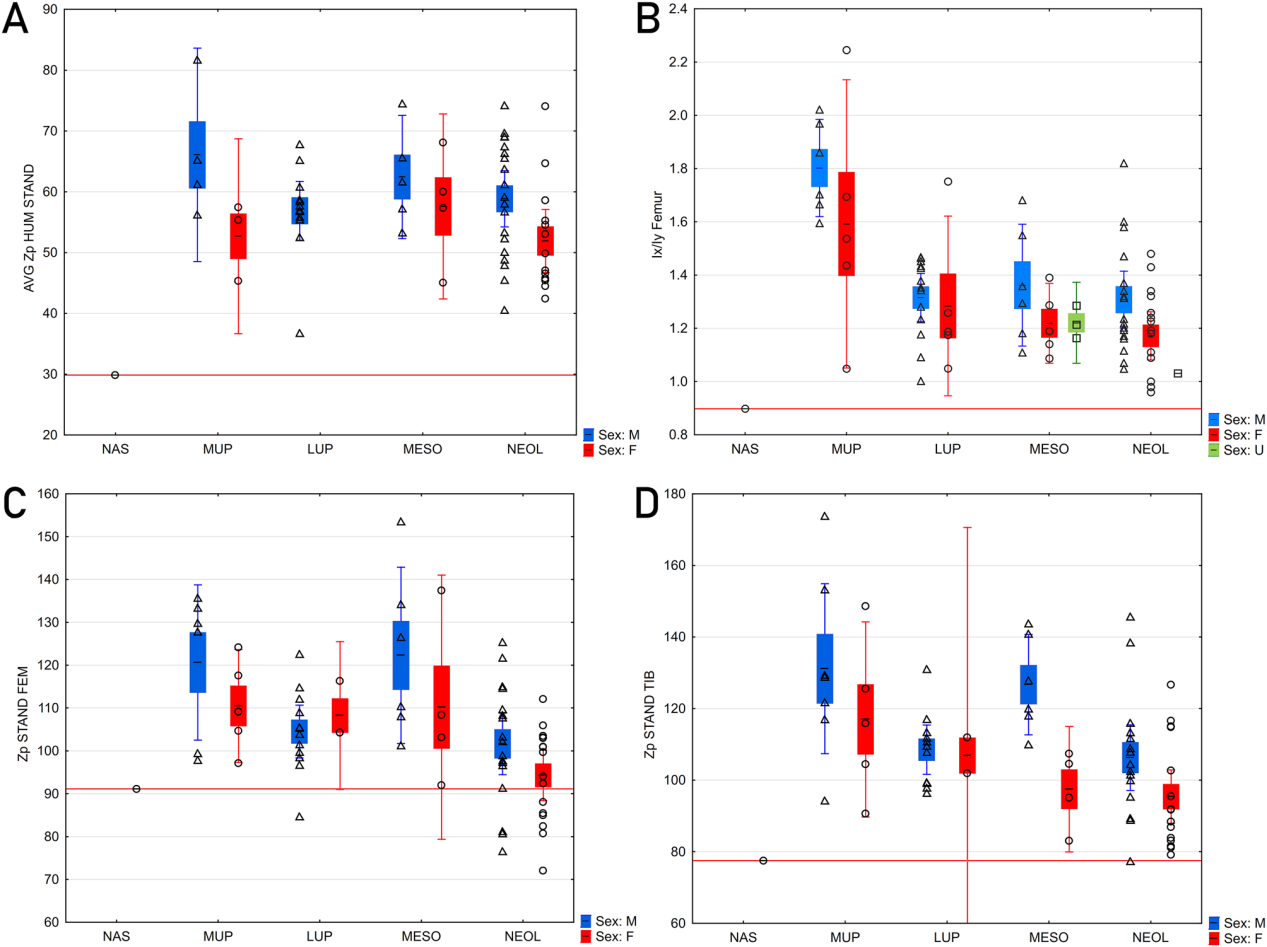
Boxplots comparing the cross-sectional geometric properties of Nasino 2 with a sample of Italian Middle Upper Paleolithic (MUP), Late Upper Paleolithic (LUP), Mesolithic (MESO), and Neolithic (NEOL) individuals, categorized by sex (raw data in SI 2). (**A**) Midshaft humeral size-standardized mechanical robusticity (average of the two sides); (**B**) Shape index of the femur I_x_/I_y_ (second moment of area about the M-L axis/second moment of area about the A-P axis); (**C**) Midshaft femoral size-standardized mechanical robusticity; (**D**) Midshaft tibial size-standardized mechanical robusticity. Boxplots indicate the mean, the standard error, and 95% confidence intervals.

While body proportions of Nasino 2 appear normal for a Mesolithic female, the mechanical robusticity of her long bones, evaluated using cross-sectional geometry (CSG^65,66^) is particularly low (Fig. 6). This is true especially for humeral robusticity, Nasino 2 being well below the range of variability of a comparative sample of Paleolithic, Mesolithic, and Neolithic individuals (raw data in Supplementary Information 3). However, the relative cortical thickness of the left humerus at midshaft is among the highest in a comparative sample of European Middle and Late Upper Paleolithic individuals (Figure S8 in Supplementary Information 3). In the lower limb, Nasino’s femoral robusticity is at the lower end of variability for prehistoric foragers, while tibial robusticity is among the lowest in the entire comparative sample (Fig. 6).

Femoral and tibial shape indices can provide information on mobility levels, high indices usually being associated with frequent and strenuous traversing of terrain^67^. The femoral shape index of Nasino 2 is below 1, indicating a buttressing in the medio-lateral axis, and is the lowest in the comparative sample (Fig. 6). Conversely, tibial cross-sectional mechanical shape of Nasino 2 is the highest in the Mesolithic sample.

## Discussion

This study provides the first multi-proxy data on a Mesolithic adult from northwestern Italy, allowing for an initial assessment of the paleoecology, paleobiology, and funerary behavior of these archaeologically elusive early Holocene foragers. Nasino cave, as well as the other nearby site that yielded Early Mesolithic human remains, Arma Veirana^20^, are only about 14 km from the current seashore, and numerous pierced *Columbella rustica* shells were included in the burials^68^. Despite this proximity and the familiarity with the sea implied by mollusk shell ornaments, the biochemistry of Nasino skeletons suggests that the exploitation of coastal resources was not the focus of Early Mesolithic subsistence in the area. In this, Nasino is similar to other Mediterranean Mesolithic sites^15,69,70^. In Italy, the best studied areas are the eastern Alps and the Emilian Apennines, where Sauveterrian groups seasonally hunted in high pastures and lowlands^16^, exploiting terrestrial animals and freshwater resources, supplemented by plant foods^6,71^. Even in the coastal sites of Sicily, marine foods became important only in the Late Mesolithic, while in the earlier phases, terrestrial resources were predominantly exploited^41,72,73^. This may be due to the lower productivity and the limited tidal range of the Mediterranean compared to the Atlantic or the western Baltic coasts ^15,74^. However, it is fundamental to obtain direct biochemical evidence on human remains because early sites bearing archaeological evidence of marine food exploitation may have been submerged by rising sea levels^75^. Indeed, the results from Nasino not only suggest an exploitation of terrestrial food, but the low nitrogen and sulfur values hint that the entire ecology of these foragers was based on the exploitation of high-altitude inland pastures, probably towards the Maritime Alps. In contrast with Mesolithic groups, Late Epigravettian hunters from the same general area (Arene Candide Cave) exploited mainly terrestrial resources closer to the Ligurian coast, as indicated by their higher nitrogen values, and the similarity of their sulfur isotopic values with those of later Neolithic individuals found at Arene Candide and Nasino caves ^52^.

Bayesian modelling of Nasino isotopic data is compatible with a diet based on ibex and freshwater resources. Interestingly, ibex constitutes up to 80% of the fauna in layers attributed to the Late Upper Paleolithic at the nearby site of Arma dello Stefanin^76,77^ (440 m a.s.l). Unfortunately, no faunal assemblage from documented Mesolithic layer is available, but warming climate in the Holocene most likely pushed the ibex to higher altitudes^78^, towards the Maritime Alps. Indeed, sediment core analysis from Pian del Lago (830 m a.s.l., eastern Liguria) highlight a marked increase in temperatures and the spread of more mesophilous tree taxa beginning around 9970 cal. BP^79^, which certainly led to a rise in the tree line and affected the distribution of fauna. It is possible that, as temperature increased, Early Holocene hunters at Nasino continued to exploit the same resource, shifting their seasonal movements towards the high mountains, and returning to the valleys of Liguria during the winter. However, it should be noted that FRUITS, like other Bayesian models, are useful to explore different scenarios, but their results depend on the resources that were initially considered for the model. Therefore, those results should not be considered as a proven fact or the only possible explanation^80^. Future research will further explore changes in subsistence-related seasonal mobility of Early Holocene Ligurian foragers through strontium and oxygen isotopic analysis, and the study of environmental cores.

The results we obtained for Nasino also suggest a novel interpretation for the low δ^15^N values of the Arma Veirana neonate, which were previously attributed to an absence of breastfeeding signals^20^. These values may now best be accounted for by the initial effect of breastfeeding in a 40–50 days-old infant belonging to a foraging group with a similar ecology to Nasino individuals.

Even though isotopic analysis depicts a scenario of significant mobility in mountainous landscapes for these Mesolithic foragers, the structural analysis of skeletal remains of Nasino 2 revealed that she was quite mechanically gracile for her size. Although biomechanical analyses should not be used to make inferences about activity levels and types of single individuals, being more appropriate to infer population-level subsistence-related patterns using large samples, they can highlight when an individual significantly deviates from the norm of the reference population. In this case, Nasino 2 is significantly more gracile when compared to other prehistoric groups, including Mesolithic Italian individuals, and is compatible with a pathological lack of subperiosteal apposition during growth^81^, which may result from a prolonged lack of activity during adolescence. Indeed, most subperiosteal growth takes place during the pre- and peri-pubertal periods^82,83^, and activity-related mechanical loadings are fundamental to attain full growth potential^65,84,85^. Lack of activity in Nasino 2 may have been the consequences of a traumatic event whose observable skeletal traces are the deformities and permanent limited motor functions of the right forearm. Chronic pain, neuropathy, and infections are not uncommon adverse outcomes of pediatric fractures of the distal forearm in modern clinical settings^58^, and one should expect them to have been much more frequent in prehistory. Metabolic insults such as malnutrition, long-term illness, reduced mechanical loadings, and compromised motor functions significantly alter bone development by slowing down subperiosteal apposition^81^ (Supplementary Information 3). The fact that Nasino 2 has normal body dimensions when compared to other Mesolithic individuals would be explained by the fact that diaphyseal cross-sectional size appears to be more sensitive to environmental factors, and less genetically canalized, than bone length and articular size^86,87^. The patterning of gracility and the shape indices in the lower limb as well appear most compatible with limited vigorous mobility for Nasino 2 during adolescence compared to other prehistoric foragers (extended discussion in Supplementary Information 3). However, these results do not necessarily suggest that Nasino 2 was inactive at the time of death: increased mechanical loading from mid-adolescence through early adulthood would have an effect mainly on the endosteal surface, leading to a contraction of the medullary cavity^88^. Conversely, prolonged inactivity and immobilization after the completion of development leads to thinner cortical bone due to medullary area expansion, with little or no change in total area^89^. The relative cortical thickness of Nasino 2 was examined at the midshaft level of the humerus, and resulted among the highest in a sample of Middle and Late Upper Paleolithic Europeans (extended discussion in Supplementary Information 3). Medullary stenosis in Nasino 2 is compatible with a vigorous use of the upper limb during adulthood, after the period of halted periosteal apposition during adolescence that determined the small subperiosteal size of the cross section.

Indeed, despite Nasino 2’s gracility and probably limited motor functions in one arm, she lived for several years after the trauma. Given our current limited knowledge about the lifeways of Early Holocene foragers in this region, it is difficult to reconstruct the life experience of this individual, to what degree her impairments constituted a disability, and whether her social group accommodated for her possible limitations^90–92^. However, her group certainly accorded her a formal funerary treatment, following a program similar to other Mesolithic burials in northern Italy (supine, with extended limbs, and surrounded by stones^18,19,21,93^). In addition, like for the infant burial of Arma Veirana, they included in her burial several pierced *Columbella rustica* shells^20,68^*.* This ornament in particular seems to have had a special role in the construction and maintenance of Mesolithic personhood and identity, and was exchanged over long distances throughout Europe ^94–96^^.^ Nasino 2’s personhood as a Mesolithic forager was therefore recognized in her funerary representation.

Overall, the multi-proxy analysis on the few skeletal remains from Nasino allowed for several new insights on Early Mesolithic foragers in Liguria, a period and region for which there was little paleoecological information. Bone biochemistry of three individuals (including the neonate from Arma Veirana) suggests a shift in ecology and settlement patterns towards higher altitudes when compared to earlier Paleolithic groups, highlighting a component of the population-level complex changes in biocultural human adaptations with the climatic transition at the Pleistocene-Holocene boundary^97^. In turn, the osteobiography of Nasino 2 provided unique glimpses into the life experience of a specific female forager, which included traumatic injuries and developmental disturbances during adolescence, followed by several years of living with an upper limb deformity and probable manipulatory impairment. On the degree of resilience and group care that was needed by this individual it is only possible to speculate at this stage; further findings are needed to provide a more complete picture of the social, ecological, and biological dynamics of the last hunter-gatherers of Liguria.

Finally, it should be noted that these results derive from renewed studies of remains that have been excavated over fifty years ago and that their importance for the reconstruction of Mesolithic lifeways in Liguria was recognized only after radiocarbon dating corrected their chrono-cultural attribution. The reanalysis of old collections and of their documentation using modern methods is a field of research that should be encouraged, because it restitutes to the scientific community skeletal remains that often were originally overlooked due to the low quality of their contextual data, by reason of poor past excavation and documentation practices^98^. This approach, although difficult and time consuming, has been proven extremely fruitful in Liguria, with the re-discovery of previously unrecognized Early Neolithic^26,27^ and even Upper Paleolithic burials^25^. It is ongoing in several other sites, including Arma dello Stefanin and Arene Candide, and is helping to resolve the apparent paradox of the relative absence of the Mesolithic in the region and its perceived implications for other phenomena, such as the Neolithic colonization in the area. It is more than likely that other European historical skeletal collections hold a similar potential.

## Materials and methods

The remains of Nasino 2 and 3 are housed at the Soprintendenza Archeologia, Belle Arti e Paesaggio per le province di Imperia e Savona (Genova, Italy). The skeletal material from Arene Candide analyzed here is housed at the Museo di Storia Naturale dell’Università di Firenze, sezione di Antropologia ed Etnologia (Florence, Italy), the Museo Archeologico del Finale (Finale Ligure, Italy), and the Museo di Archeologia Ligure (Genova, Italy). Soprintendenza Archeologia, Belle Arti e Paesaggio per le province di Imperia e Savona provided permission for the study of the material.

### Biological profile, osteometrics, biomechanics, paleopathology

Age at death for the Nasino individual and comparative sample was estimated using standard methods including the auricular surface of the ilium^99,100^, and dental and skeletal development for juveniles^36,59^.Standard methods were employed for the determination of the biological sex^101–103^.

Evidence of trauma in the right forearm of Nasino 2 was investigated via biplanar radiographs using an Agfa DXD40_1000C at the medical center “Casa della Salute” of Genova Multedo.

Nasino 2 skull, os coxae, and long bones (humeri, radii and ulnae, left femur and left tibia) were surface-scanned in 3D, using the DAVID SLS-3 structured light scanner (David Group 2007–2015, now property of HP). The models were uploaded in the online repository Morphosource (www.morphosource.org project “The Mesolithic human remains from Arma di Nasino, Liguria, Italy”) and are downloadable from the scientific community for the purpose of research upon request directly from the website.

The method used to evaluate the mechanical competence of Nasino 2 long bones (humeri, femur, and tibia) is called Cross-Sectional Geometry (CSG^65,104^), and the data collection employed the “Solid CSG” method^105^. Biomechanical robusticity (i.e. diaphyseal rigidity scaled by body size) was calculated from the shape and size of the cross-sections at specific levels of the diaphysis (mechanical length, as indicated in Ruff^106^), using the polar moment of area as a measure of overall bending and torsional rigidity. Results were scaled by body size using bone mechanical length and estimated body mass^107^. To minimize the length of this methods section, the description of the CSG method and an extended results and discussion section on the mechanical properties of Nasino 2 is provided in Supplementary Information 3. Comparative data for CSG and osteometric analysis derives from the literature and previous research by the authors and is available, together with the raw data of Nasino 2, osteometric measurements drawn and re-checked from previous research^35^, and bibliographic references, in Supplementary Information 3.

### Radiocarbon dating and isotopic analysis

The bone collagen used for both elemental composition analysis and radiocarbon dating (GrM-13521 and GrM-13522) was extracted at the LAMPEA biochemical platform (UMR 7269, Aix-en-Provence, France), by using the protocol on chunk. Samples were abraded with aluminum oxide by a sandblaster to remove the external cortical surface of the bone. The clean sample was then demineralized in HCl (0.05 M) at 4 °C for several days and rinsed with distilled water after demineralization was completed. Samples were then cleaned in NaOH for 20°h to remove potential remaining contaminant, rinsed and solubilized in HCl (0.01 M) at 70 °C for 24 h. Solubilized collagen was filtered with EzeeFilter® device. Each sample was then frozen and freeze-dried during 24 h. The collagen was analyzed by a Europa Scientific EA analyzer (IsoAnalytical Ltd, Crewe, UK) to check the quality control. The preservation of collagen and reliability of isotopic data were controlled according to international recommendations: C ≥ 30%, N ≥ 10%^108^, and C/N between 2.9 and 3.6^109^. The laboratory standards used (for CN: IA-R042 bovine liver; IA-R001 wheat flour; IA-R005/IA-R045 mixture of beet sugar and ammonium sulfate. For S: IA-R061 IAEA-SO-5 barium sulfate, IA-R068 soy protein and IA-R069 tuna protein) were calibrated against IAEA international standards for all measurements; the measurement error is 0.1‰ for carbon and nitrogen and 0.2‰ for sulfur. Comparative data for the isotopic analysis are derived from the literature; these data and the bibliographic references are available in Supplementary Information 2.

At the Centre for Isotope Research (CIO), University of Groningen, in total five different Nasino samples have been measured on stable isotopes (^13^C and ^15^N) and radiocarbon. Two samples (GrM-13521 and GrM-13522) were submitted as collagen sample (pretreated at the LAMPEA biochemical platform as described above). The other three samples (GrM-15944, GrM-21897 and GrM-21898) were submitted as bone samples and were pretreated at CIO to collagen. The applied collagen preparation method at CIO was “ABA-Longin”. First the material was treated with 4% HCl, 1% NaOH and 4% HCl at room temperature respectively (rinsing with decarbonized water to neutral pH between each step). Then the collagen fraction was dissolved in decarbonized water at 80 °C and pH3 for one night. The obtained solution was filtered over a 50 μm filter and then dried in an oven at 80 °C for at least 24 h. Maximal 5.5 mg collagen was weighed in tin capsules and combusted to CO_2_ with an Elemental Analyzer (Elementar Vario Isotope Cube^TM^). %C, %N and C/N were measured to check the quality of the collagen. A small part of the CO_2_ was measured on stable isotopes with an IRMS (δ^13^C and δ^15^N; IsoPrime 100^TM^). The main part of the CO_2_ was trapped cryogenically in flasks and then graphitized to solid carbon in a graphitization system (at 600 °C, adding H_2_ and using Fe powder as catalyst). On the day of AMS measurement, the graphite is pressed in an AMS-target for measurement. An overview of the applied methods is also given in Dee et al.^110^. For ^14^C measurement, the ^14^C/^12^C and ^13^C/^12^C ratios are measured at CIO using a 200 kV IonPlus MICADAS AMS system^111,112^. Several quality control samples are pretreated and measured (IRMS and AMS) to verify the calibration of the results and whether no failures have been made during the lab-process. Every pretreatment batch includes a known age sample and/or duplicate of a former pre-treated sample. Both these quality assurance samples undergo the same pre-treatment as the other samples of the same material in the relevant batch. Each combustion batch also contains a series of calibration standards (Oxalic acid-II), background material (material with a ^14^C value below the detection limit) and various known-age reference materials. All ^14^C samples in the IC batch are measured in the same AMS-batch. The ^14^C measurement results are calculated to year BP, including background correction and isotope fractionation correction (using δ^13^C measured with AMS), according to the conventions^113,114^. Based on the measurement results of the quality assurance samples and reference standards, it is assessed whether the preparations, graphitization and measurement of the various samples in the measurement batch have been proceeded well. If deviations are found, reanalysis takes place (starting from the step in the lab process where the deviation had occurred). The reported measurement uncertainty includes beside the AMS-measurement error for the particular sample, also a variation as observed in multiple measurements of a particular sample material. The long-term performance and accuracy of the AMS measurements at CIO since September 2017 (start with MICADAS) are summarized in Aerts-Bijma et al.^115^. So far, CIO joined the main inter-comparison rounds for ^14^C labs in which different materials of different ages are measured and compared between different ^14^C labs^116^. One of the bones measured in one of these rounds, a bone sample called VIRI-F, is regularly pretreated to collagen and measured by CIO (in the time period in which the Nasino bones were also pretreated). The average ^14^C age is 2539 ± 34 years BP (average and standard deviation in n = 18 results), where the consensus value is set to 2513 ± 5 years BP^116^.

### Supplementary Information

Supplementary Information 1.

Supplementary Information 2.

Supplementary Information 3.

## Data Availability

The datasets generated during and/or analyzed during the current study are available from the corresponding author on reasonable request.
