# Author Correction: Human remains from Arma di Nasino (Liguria) provide novel insights into the paleoecology of early Holocene foragers in northwestern Italy

**DOI:** 10.1038/s41598-023-46311-9

**Published:** 2023-11-13

**Authors:** Vitale S. Sparacello, Gwenaëlle Goude, Alessandra Varalli, Irene Dori, Claudine Gravel-Miguel, Julien Riel-Salvatore, Sanne W. L. Palstra, Jacopo Moggi-Cecchi, Fabio Negrino, Elisabetta Starnini

**Affiliations:** 1 Dipartimento di Scienze della Vita e dell’Ambiente, Sezione di Neuroscienze e Antropologia, Università degli Studi di Cagliari, Cagliari, Italy; 2 CNRS, Aix Marseille Univ, Minist Culture, LAMPEA, UMR 7269, 5 rue du Château de l’Horloge, 13097 Aix-en-Provence, France; 3 CASEs Research Group, Department of Humanities, Universitat Pompeu Fabra, Barcelona, Spain; 4 Dipartimento di Biologia, Università degli Studi di Firenze, Florence, Italy; 5 Département d’Anthropologie, Université de Montréal, Montréal, QC Canada; 6 Center for Applied Fire and Ecosystem Science, New Mexico Consortium, Los Alamos, NM USA; 7 Centre for Isotope Research, ESRIG, University of Groningen, Groningen, The Netherlands; 8 DAFIST - Dipartimento di Antichità, Filosofia, Storia, Università degli Studi di Genova, Genoa, Italy; 9 Dipartimento di Civiltà e Forme Del Sapere, Università di Pisa, Pisa, Italy

Correction to: *Scientific Reports* 10.1038/s41598-023-40438-5, published online 29 September 2023

The original version of this Article contained errors. In the original version of this Article, Supplementary Information File 1 was incorrectly cited as “Supplementary Figure S1”. In addition, the labels to the accompanying Supplementary Information 2 and Supplementary Information 3 were incorrectly given as Supplementary Information 3 and Supplementary Information 2.

Consequently, in the Results section:

Where

“A few bones of a third individual (Nasino 3), not in anatomical connection, were found in the northwestern corner of the cave against the wall (Supplementary Information Figure S1)”

now reads

“A few bones of a third individual (Nasino 3), not in anatomical connection, were found in the northwestern corner of the cave against the wall (Supplementary Information 1)”

where

“We therefore investigated the diet of the two adult Mesolithic individuals by comparing them with earlier Upper Paleolithic foragers that lived in the same area: the Late Epigravettian individuals (n = 13) from the Arene Candide Cave necropolis, dated between 12,800–12,500 and 12,100–11,800 cal. BP25, analyzed for this study, and the published data for the Gravettian “Il Principe” (“The Prince”) from Arene Candide Cave, dated to ca. 27,900–27,300 cal. BP37 (Fig. 2A; raw data in Supplementary Information 1).”

now reads

“We therefore investigated the diet of the two adult Mesolithic individuals by comparing them with earlier Upper Paleolithic foragers that lived in the same area: the Late Epigravettian individuals (n = 13) from the Arene Candide Cave necropolis, dated between 12,800–12,500 and 12,100–11,800 cal. BP25, analyzed for this study, and the published data for the Gravettian “Il Principe” (“The Prince”) from Arene Candide Cave, dated to ca. 27,900–27,300 cal. BP37 (Fig. 2A; raw data in Supplementary information 2).”

where

“To investigate this possibility, we compared the results obtained for Nasino with published data for wild herbivores from Mesolithic and Upper Paleolithic sites near the Mediterranean Coast (Sicily, Corsica, and Croatia) and from the Italian Alps (Veneto and Trentino regions; raw data in Supplementary Information 1).”

now reads

“To investigate this possibility, we compared the results obtained for Nasino with published data for wild herbivores from Mesolithic and Upper Paleolithic sites near the Mediterranean Coast (Sicily, Corsica, and Croatia) and from the Italian Alps (Veneto and Trentino regions; raw data in Supplementary Information 2).”

where

“Isotopic values for targeted animal resources, targets and information about the model data implemented are provided in Supplementary Information 1.”

now reads

“Isotopic values for targeted animal resources, targets and information about the model data implemented are provided in Supplementary Information 2.”

where

“Considering the femoral head superoinferior diameter as a proxy for body mass, and femoral maximum length as a proxy for stature, Nasino 2 shows body proportions that are more compatible with the Italian Mesolithic sample than with the Ligurian Neolithic females, or with Pleistocene humans (detailed analysis in Supplementary Information 2).”

now reads

“Considering the femoral head superoinferior diameter as a proxy for body mass, and femoral maximum length as a proxy for stature, Nasino 2 shows body proportions that are more compatible with the Italian Mesolithic sample than with the Ligurian Neolithic females, or with Pleistocene humans (detailed analysis in Supplementary Information 3).”

where

“This is true especially for humeral robusticity, Nasino 2 being well below the range of variability of a comparative sample of Paleolithic, Mesolithic, and Neolithic individuals (raw data in Supplementary Information 2). However, the relative cortical thickness of the left humerus at midshaft is among the highest in a comparative sample of European Middle and Late Upper Paleolithic individuals (Figure S8 in Supplementary Information 2). In the lower limb, Nasino’s femoral robusticity is at the lower end of variability for prehistoric foragers, while tibial robusticity is among the lowest in the entire comparative sample (Fig. 6).”

now reads

“This is true especially for humeral robusticity, Nasino 2 being well below the range of variability of a comparative sample of Paleolithic, Mesolithic, and Neolithic individuals (raw data in Supplementary Information 3). However, the relative cortical thickness of the left humerus at midshaft is among the highest in a comparative sample of European Middle and Late Upper Paleolithic individuals (Figure S8 in Supplementary Information 3). In the lower limb, Nasino’s femoral robusticity is at the lower end of variability for prehistoric foragers, while tibial robusticity is among the lowest in the entire comparative sample (Fig. 6).”

where

“Metabolic insults such as malnutrition, long-term illness, reduced mechanical loadings, and compromised motor functions significantly alter bone development by slowing down subperiosteal apposition^81^ (Supplementary Information 2). The fact that Nasino 2 has normal body dimensions when compared to other Mesolithic individuals would be explained by the fact that diaphyseal cross-sectional size appears to be more sensitive to environmental factors, and less genetically canalized, than bone length and articular size^86,87^. The patterning of gracility and the shape indices in the lower limb as well appear most compatible with limited vigorous mobility for Nasino 2 during adolescence compared to other prehistoric foragers (extended discussion in Supplementary Information 2).”

now reads

“Metabolic insults such as malnutrition, long-term illness, reduced mechanical loadings, and compromised motor functions significantly alter bone development by slowing down subperiosteal apposition^81^ (Supplementary Information 3). The fact that Nasino 2 has normal body dimensions when compared to other Mesolithic individuals would be explained by the fact that diaphyseal cross-sectional size appears to be more sensitive to environmental factors, and less genetically canalized, than bone length and articular size^86,87^. The patterning of gracility and the shape indices in the lower limb as well appear most compatible with limited vigorous mobility for Nasino 2 during adolescence compared to other prehistoric foragers (extended discussion in Supplementary Information 3).”

and where

“The relative cortical thickness of Nasino 2 was examined at the midshaft level of the humerus, and resulted among the highest in a sample of Middle and Late Upper Paleolithic Europeans (extended discussion in Supplementary Information 2).”

now reads

“The relative cortical thickness of Nasino 2 was examined at the midshaft level of the humerus, and resulted among the highest in a sample of Middle and Late Upper Paleolithic Europeans (extended discussion in Supplementary Information 3).”

Additionally, in the Materials and Methods section, where

“To minimize the length of this methods section, the description of the CSG method and an extended results and discussion section on the mechanical properties of Nasino 2 is provided in Supplementary Information 2. Comparative data for CSG and osteometric analysis derives from the literature and previous research by the authors and is available, together with the raw data of Nasino 2, osteometric measurements drawn and re-checked from previous research35, and bibliographic references, in Supplementary Information Table 2.”

now reads

“To minimize the length of this methods section, the description of the CSG method and an extended results and discussion section on the mechanical properties of Nasino 2 is provided in Supplementary Information 3. Comparative data for CSG and osteometric analysis derives from the literature and previous research by the authors and is available, together with the raw data of Nasino 2, osteometric measurements drawn and re-checked from previous research35, and bibliographic references, in Supplementary Information 3.”

and where

“Comparative data for the isotopic analysis are derived from the literature; these data and the bibliographic references are available in Supplementary Information 1.”

now reads

“Comparative data for the isotopic analysis are derived from the literature; these data and the bibliographic references are available in Supplementary Information 2.”

The original Article and the Supplementary information file names have been corrected.

