# Supplementary figures and images for: Human remains from Arma di Nasino (Liguria) provide novel insights into the paleoecology of early Holocene foragers in northwestern Italy
Source: Sci Rep. 2023 Sep 29;13:16415. doi: 10.1038/s41598-023-40438-5 (PMC10541424; doi:10.1038/s41598-023-40438-5)

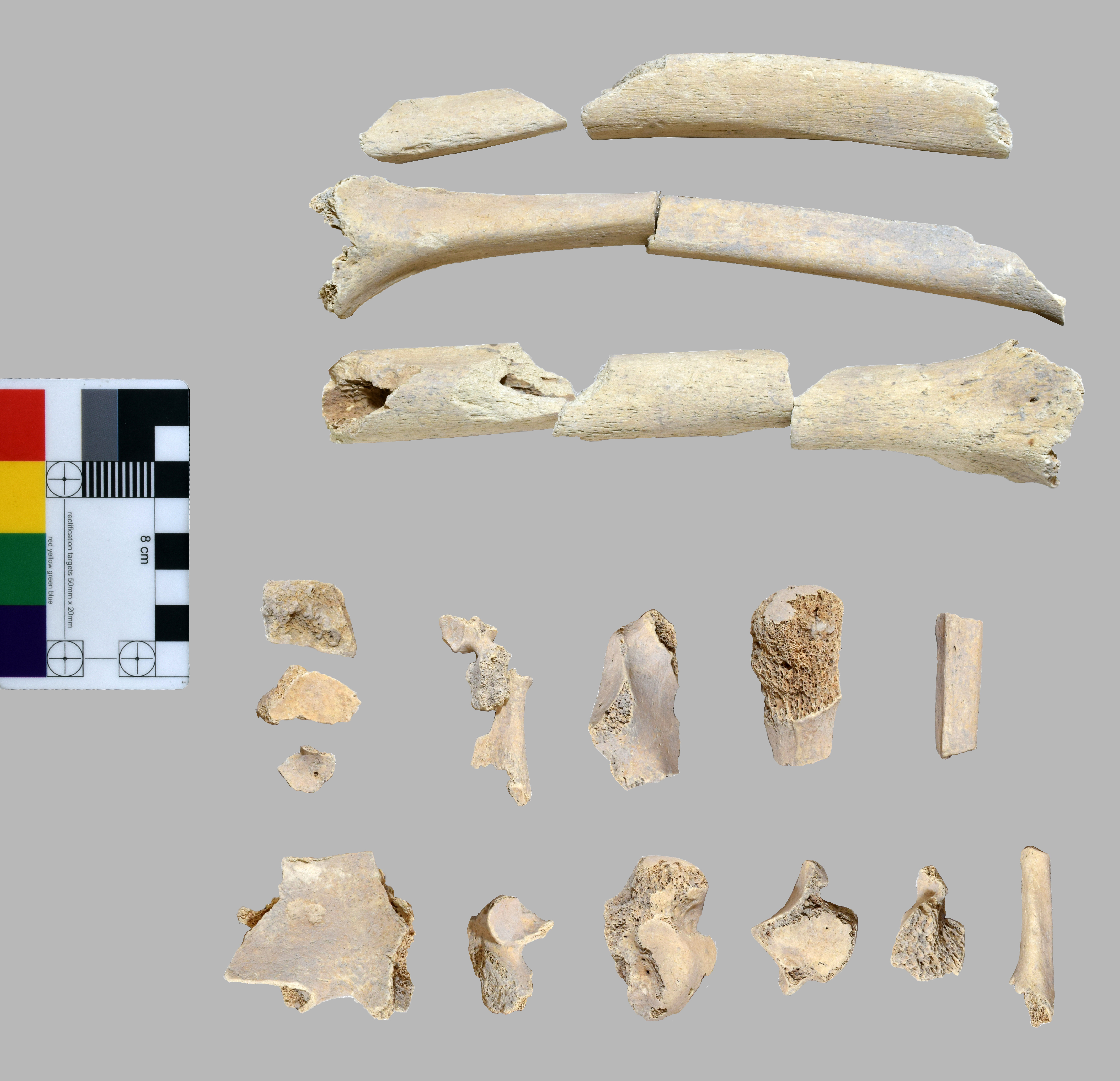

Supplement: Supplementary file 1 — Supplementary Information 1. [file 41598_2023_40438_MOESM1_ESM.jpg]
