# Supplementary material for: Human remains from Arma di Nasino (Liguria) provide novel insights into the paleoecology of early Holocene foragers in northwestern Italy
Source: Sci Rep. 2023 Sep 29;13:16415. doi: 10.1038/s41598-023-40438-5 (PMC10541424; doi:10.1038/s41598-023-40438-5)
Supplement: Supplementary file 3 — Supplementary Information 3. [file 41598_2023_40438_MOESM3_ESM.zip › Supplementary Information 3 - CSG/SI 3 CSG extended analysis and discussion.docx]

**Supplementary Information 3 – Extended CSG analysis and discussion**

1. **Cross-sectional geometry introduction**

The biomechanical analysis of postcranial functional adaptations via cross-sectional geometry (CSG) is based on the widely accepted notion that bone tissue optimizes to its mechanical environment to maintain physiological strains within normal limits (“Wolff’s Law”, better referred to as “bone functional adaptation”; Pearson and Lieberman, 2004; Ruff et al., 2006b). According to this rationale, bone tissue is deposited in the shaft’s cross-section where mechanical loads require it to prevent strains exceeding the elastic limit, whereas below a certain strain threshold, the bone tissue is reabsorbed. Therefore, by analyzing the cross-sections of the diaphysis, it is possible to quantify variables that correlate with torsional bone rigidity (polar moment of area: J). This method is supported by recent experimental evidence on modern athletes (Shaw and Stock, 2009a,b, 2013; Macintosh and Stock, 2019), but there are still several factors, in addition to activity (e.g. age, diet, body proportions, and genetic makeup), that act on bone modeling and remodeling in a complex manner. Indeed, several scholars warned against a simplistic interpretation of the results (Pearson and Lieberman, 2004; Meyer et al., 2011; Jurmain et al., 2012), and pointed out inconsistencies in the model (Pearson et al., 2014). Cross-sectional mechanical properties seem to mostly reflect levels of activity during the pre- and peri-pubertal periods, while the extent of the remodeling due to activity later in life is debated (Lazenby, 1990; Pearson and Lieberman, 2004; Ruff et al., 2006b). In addition, senescence tends to enlarge long bones’ sections, and therefore CSG properties (especially in males; Martin and Atkinson, 1977; Ruff and Hayes, 1988). These limitations should have a relatively minor impact when reconstructing habitual activity and subsistence modalities of bioarchaeological samples, since it is assumed that individuals in pre-industrial societies participated to subsistence activities beginning from late childhood, and did not die at an advanced level of senescence.

Therefore, in the prehistoric bioarchaeological context, some level of correspondence between CSG properties and activity levels and types is generally accepted, once the effect of body size is factored out to obtain a measure of “robusticity” (Ruff et al., 2006b). Integration of quantitative data derived from CSG with archaeological information has therefore been widely used to draw inferences about past subsistence strategies, degrees of mobility and other habitual activities (Pinhasi and Stock, 2011; Carlson and Marchi, 2014; Sparacello et al., 2015). It should be noted that the CSG properties of a single individual may not be representative of the mechanical adaptations of a population, and therefore inferences about habitual activities of Ligurian Mesolithic people should not be based on Nasino 2. The purpose of this analysis is investigating how Nasino compares to the mechanical properties of Late Pleistocene and Holocene foragers from Italy.

1. **Methods**

Nasino 2 long bones were scanned in 3D, using the DAVID SLS-3 structured light scanner (David Group 2007-2015, now property of HP), and cross-sections were reconstructed (35% and 50% from the distal end for the humeri, and 50% for the femur and tibia) from the surface scans, which were positioned virtually according to the reference planes following Ruff (2002). The cross-sections were obtained using the “slice” function in Netfabb Standard 2018 for PC (copyright Autodesk 2017), and the CSG properties calculated using a version of the SLICE program (Nagurka and Hayes, 1980) adapted as a macro routine inserted in Scion Image release Beta 4.03. The “Solid CSG” method was used to estimate actual CSG properties from the periosteal contour via regression equations (provided in Marchi et al., 2011), as justified in previous research (Stock and Shaw, 2007; Sparacello and Pearson, 2010; Macintosh et al., 2013). All raw CSG data are available in Supplementary Information 3 Table. Solid cross sections of Nasino 2 are reproduced in Figure S1.


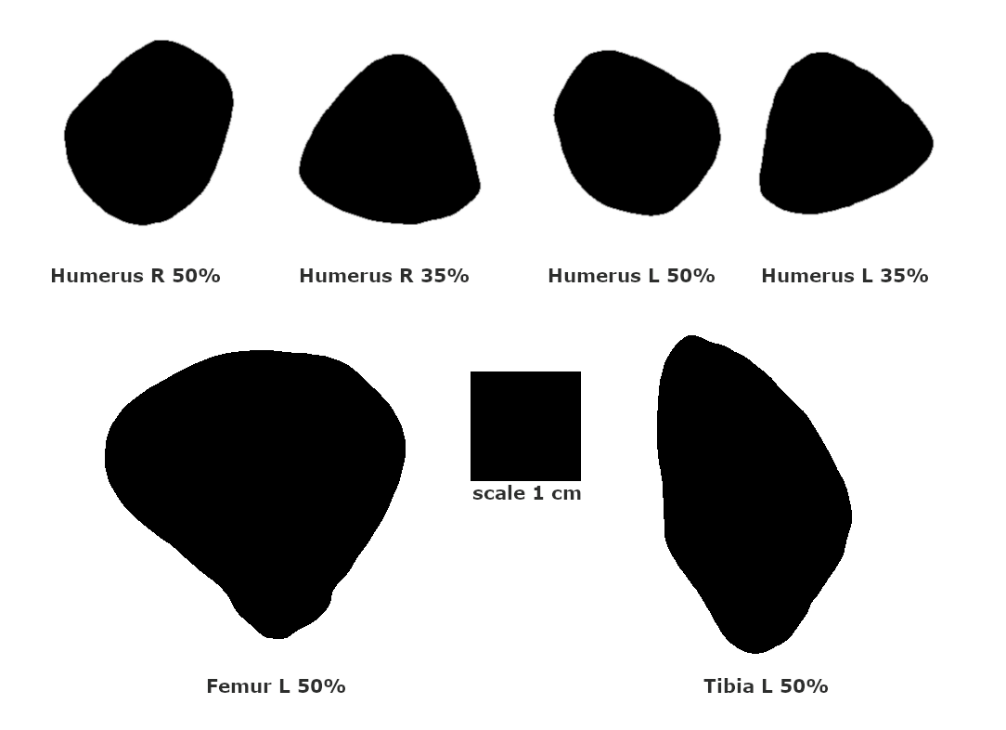


Figure S1 – Solid cross-sections of Nasino 2

The variable used to quantify overall mechanical bone strength at a given diaphyseal level is the modulus Zp section (torsional and (twice) average bending strength; Ruff, 2000, 2018). True section moduli are calculated by dividing the polar second moment of area J (torsional and (twice) average bending rigidity of the beam) by the distance from the centroid section for torsion to the outermost fiber of the section (Ruff, 2000). As done in previous research, we approximated Zp by raising J to the power of 0.73 (Ruff, 2000, 2018).

The mechanical loading on long bones is a function of physical activity, bone length and body mass (Ruff, 2000). To obtain a measure of residual strength or “robusticity”, which is assumed to correlate with the effects of activity, the estimate of overall bone strength Zp was scaled for size by dividing by mechanical bone length (as defined in Ruff, 2002) and body mass (Ruff, 2000). Body mass was estimated from the supero-inferior diameter of the femoral head following the guidelines in Trinkaus and Ruff (2012). Osteometric measurements of Nasino 2 are available in Supplementary Information Table 3 and are mostly drawn and re-checked from the published description of the material (Scarsini and Messeri, 1974). Certain osteometric measurements for the determination of the appropriate level of the cross-section and for standardization had to be estimated via regression equations based on a coeval sample, as commonly done in studies of this kind (e.g. Trinkaus and Ruff, 2012; Sparacello et al., 2017, 2020; Villotte et al., 2017; Varalli et al., 2020). The regression equations for each estimated measurement are provided in Supplementary Information 3 Table.

In order to characterize the prevalent use of one arm, the degree of humeral bilateral asymmetry in J was calculated using the formula [(JH – JL)/JL]×100 (where JH and JL are the higher and lower values of J between the two humeri, respectively) and expressed as a percentage, following previous studies (Rhodes and Knüsel, 2005; Sparacello et al., 2011). The resulting value represents an absolute (non-directional) asymmetry. Asymmetry was calculated from the absolute values of J (i.e. not standardized by body size), because any prior size standardization would be canceled.

Given their correspondence with mobility levels (Shaw and Stock, 2009a; Macintosh and Stock, 2019; Holt, 2003), lower limb CSG shape indices were also analyzed. For the femur, the ratio between Ix (second moment of area in the anteroposterior plane) and I_y_ (second moment of area in the mediolateral plane) was considered. For the tibia, the ratio of I_max_ (maximum second moment of area) to I_min_ (minimum second moment of area) was used.

Nasino CSG properties were compared with a large sample of Italian Gravettian (Middle Upper Paleolithic; c. 30-18,000 BCE), Epigravettian (Late Upper Paleolithic; c. 18-10,000 BCE), and Mesolithic (c. 10-6000 BCE) individuals, and with Neolithic people from Liguria (c. 6000-4000 BCE). Comparative data was obtained from the literature and from previous research of the authors (references in in Supplementary Information Tables A).

**Results**

Pleistocene and Holocene humans in Europe are characterized by different body size and proportions, with Middle Upper Paleolithic people retaining a tall and narrow body shape, followed by a dramatic reduction in stature with the Late Upper Paleolithic, and the prevalence of stockier proportions in the Neolithic (Churchill, 1994; Holliday, 1995, 1997, 2002; Formicola and Franceschi, 1996; Pearson, 1997; Formicola and Giannecchini, 1999; Ruff et al., 2006a). In Figure S2, the femoral head superoinferior diameter (as a proxy for body mass) is plotted on femoral maximum length (as a proxy for stature) for Nasino 2 and the comparative sample. Although there is some overlap among groups, Nasino 2 shows body proportions that are more compatible with the Mesolithic sample than with Ligurian Neolithic females, with a body mass of ca. 62 kg (Trinkaus and Ruff, 2012) and a stature of ca. 152.5 cm (applying the regression equations in Formicola and Franceschi, 1996).


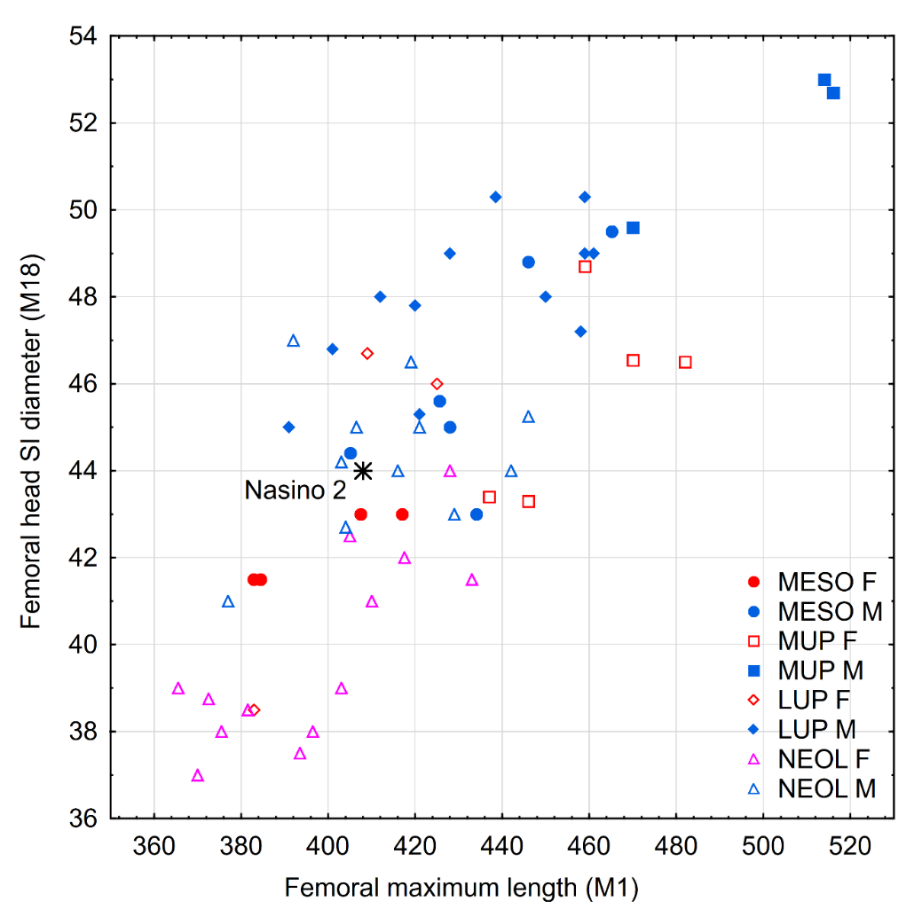


Figure S2 – Scatterplot of two femoral measurements used as a proxy for body proportions: maximum length (Martin’s M1) as a proxy for stature, and vertical head diameter (Martin’s M18) as a proxy for body mass. Nasino 2 is compared with a sample of Italian Middle Upper Paleolithic (MUP), Late Upper Paleolithic (LUP), Mesolithic (MESO), and Neolithic (NEOL) individuals.

In contrast with body proportions, the level of robusticity (Zp scaled to body size) of Nasino 2 long bones is low, especially in the humerus, for which this individual is well below the range of variability of the comparative samples (Figure S3). In contrast, Nasino’s humeral bilateral asymmetry is the highest in the Mesolithic sample (Figure S4). In the lower limb, Nasino’s femoral robusticity is at the lower end of variability for prehistoric foragers (Figure S5), while tibial robusticity is among the lowest in the entire comparative sample (Figure S6).

Shape indices of the lower limb show a contrasting pattern: Nasino’s femoral I_x_/I_y_ is below 1, indicating a buttressing in the medio-lateral axis, and is the lowest in the comparative sample (Figure S7a). Conversely tibial I_max_/I_min_ of Nasino 2 is the highest in the Mesolithic sample.


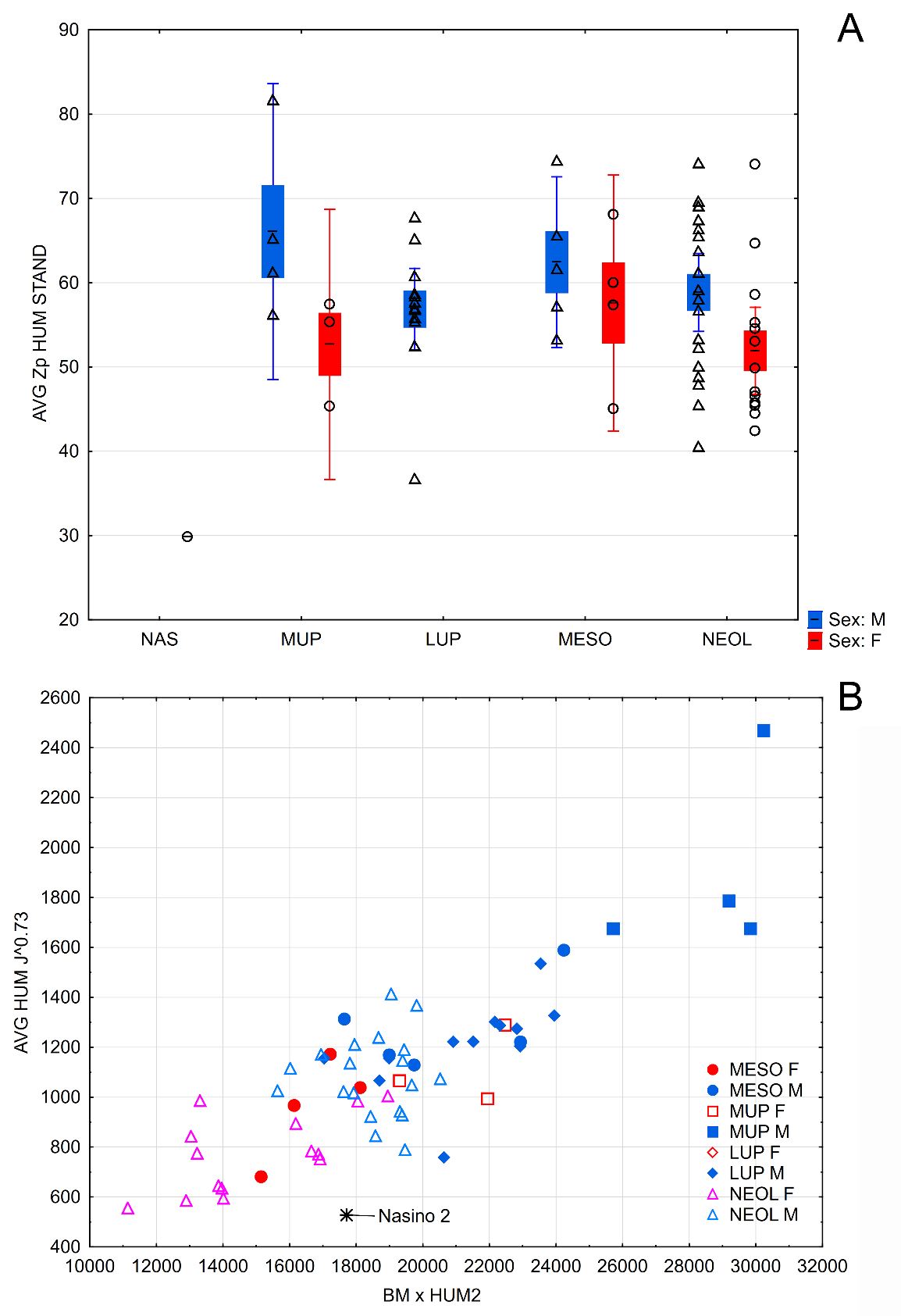


Figure S3 – A) Categorized boxplots for the section modulus of the humerus (Zp; average between sides) standardized by body size (body mass times bone length) of Nasino 2 (NAS) compared with a sample of Italian Middle Upper Paleolithic (MUP), Late Upper Paleolithic (LUP), Mesolithic (MESO), and Neolithic (NEOL) individuals. The box/whisker indicates the mean, the standard error, and the 95% confidence interval. B) Scatterplot of the section modulus of the humerus on body mass times bone length.


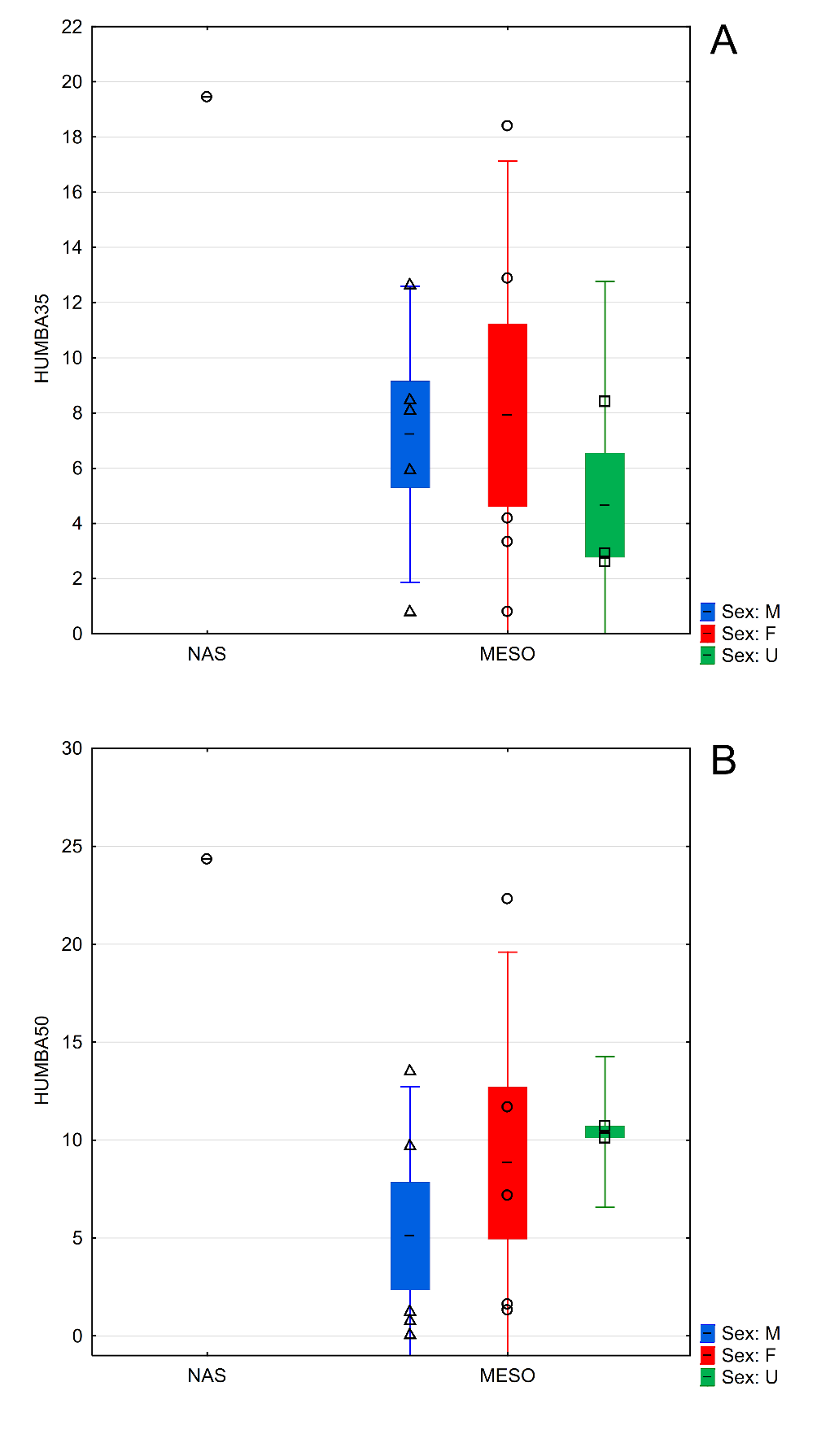


Figure S4 –Boxplots for the humeral bilateral asymmetry of Nasino 2 when compared to the Italian Mesolithic sample: A) 35% level of the diaphysis (mid-distal); B) 50% level of the diaphysis (midshaft). The box/whisker indicates the mean, the standard error, and the 95% confidence interval.


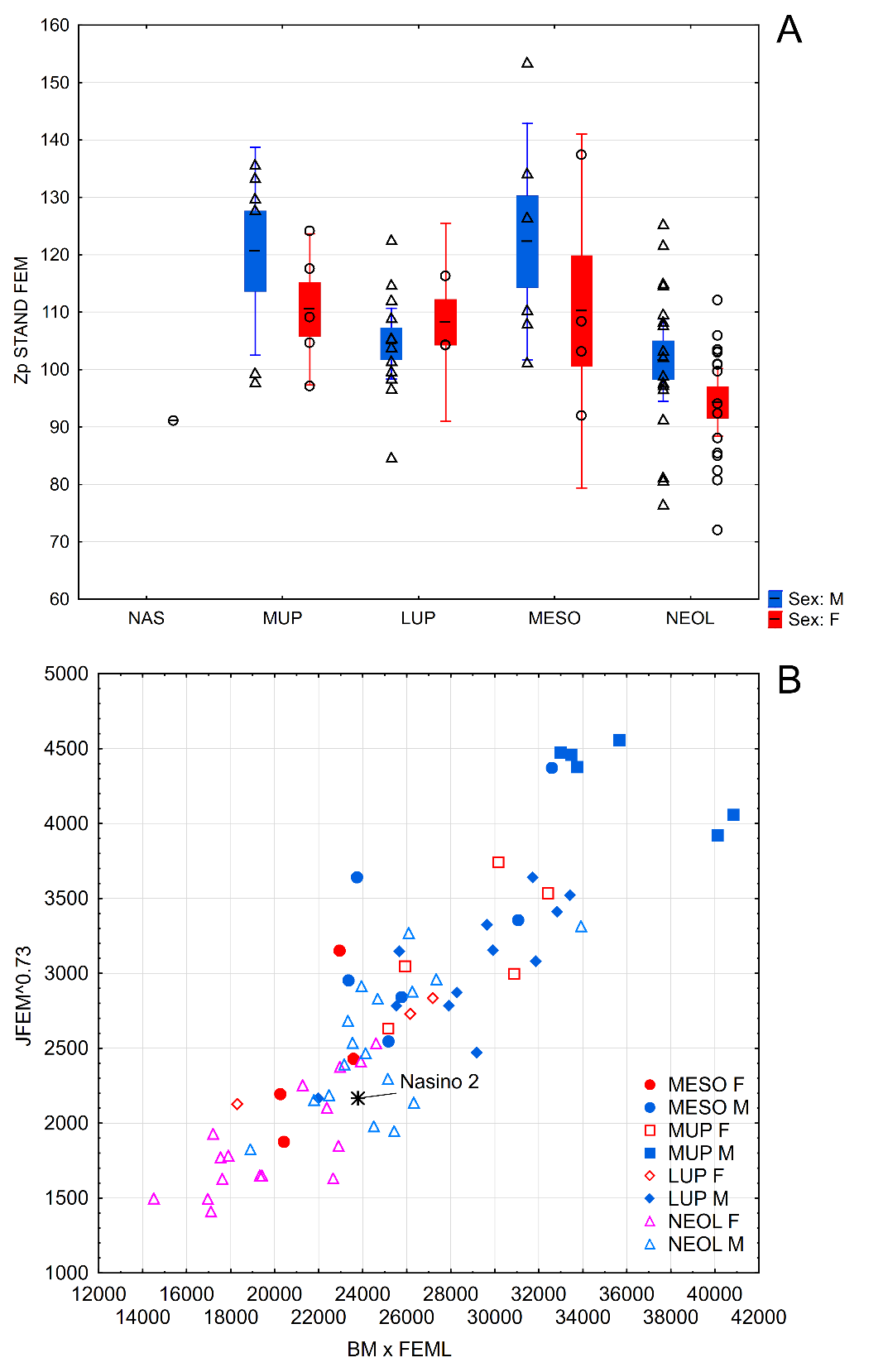


Figure S5 – A) Categorized boxplots for the section modulus of the femur (Zp) standardized by body size (body mass times bone length) of Nasino 2 (NAS) compared with a sample of Italian Middle Upper Paleolithic (MUP), Late Upper Paleolithic (LUP), Mesolithic (MESO), and Neolithic (NEOL) individuals. The box/whisker indicates the mean, the standard error, and the 95% confidence interval. B) Scatterplot of the section modulus of the femur on body mass times bone length.


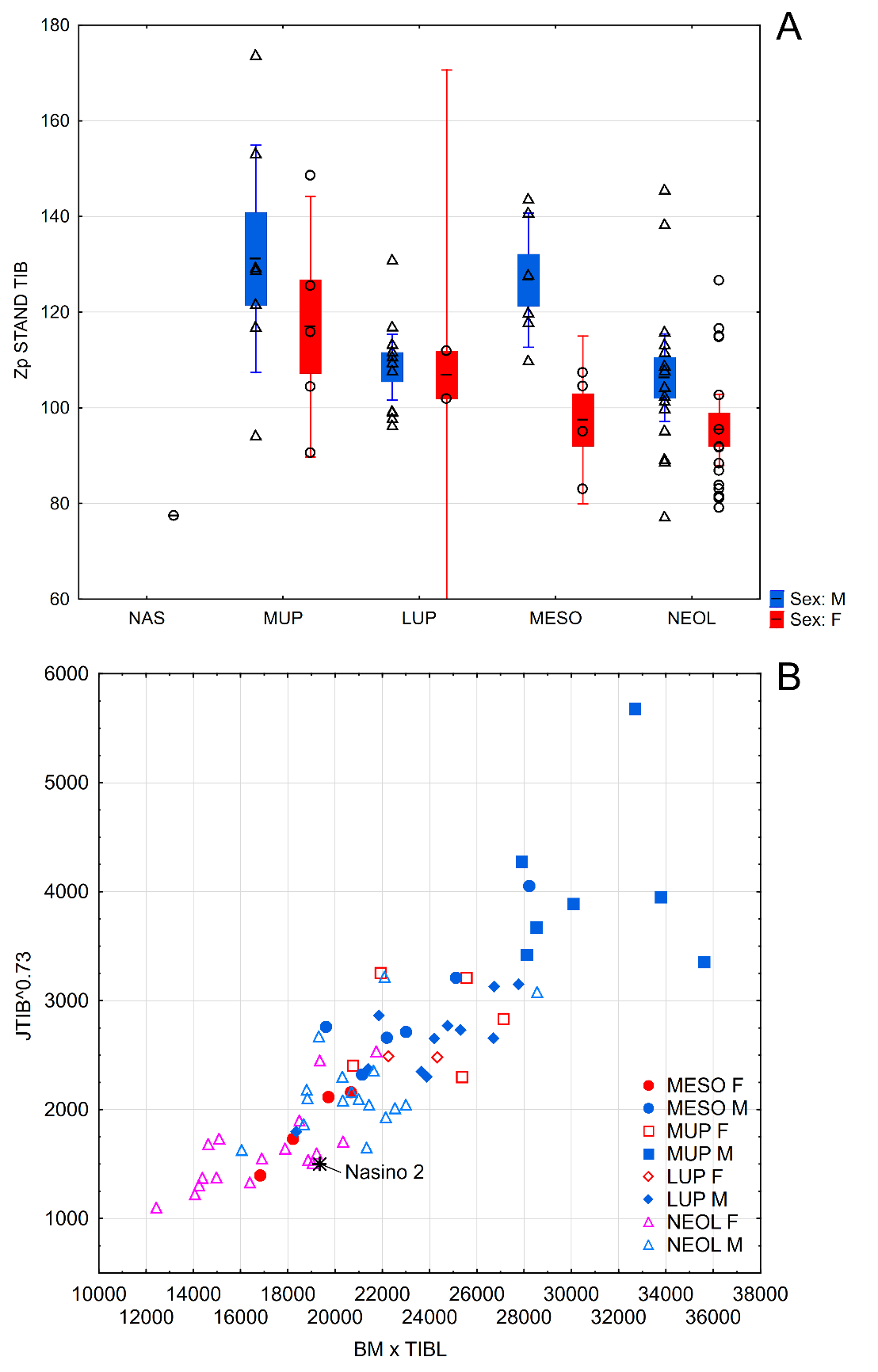


Figure S6 – A) Categorized boxplots for the section modulus of the tibia (Zp) standardized by body size (body mass times bone length) of Nasino 2 (NAS) compared with a sample of Italian Middle Upper Paleolithic (MUP), Late Upper Paleolithic (LUP), Mesolithic (MESO), and Neolithic (NEOL) individuals. The box/whisker indicates the mean, the standard error, and the 95% confidence interval. B) Scatterplot of the section modulus of the tibia on body mass times bone length.


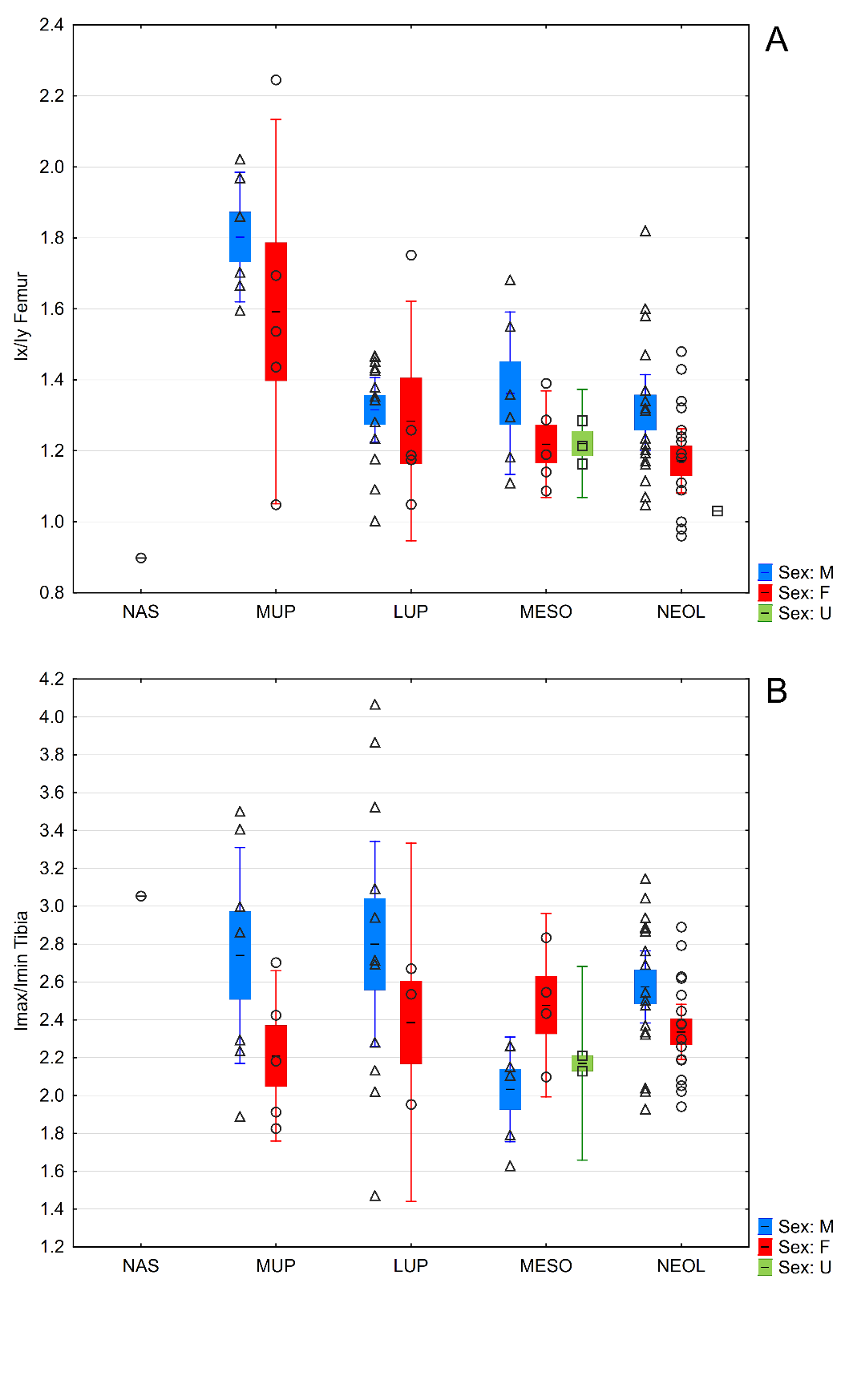


Figure S7 – A) Cross-sectional shape indices of the femur (I_x_/I_y_) of Nasino 2 (NAS) compared with a sample of Italian Middle Upper Paleolithic (MUP), Late Upper Paleolithic (LUP), Mesolithic (MESO), and Neolithic (NEOL) individuals. The box/whisker indicates the mean, the standard error, and the 95% confidence interval. B) Cross-sectional shape indices of the tibia (I_max_/I_min_).

**Discussion**

The analysis of long bone functional adaptations is usually applied to the reconstruction of activity patterns in the past, and is therefore based on a sample of individuals, ideally as large as possible in order to be representative of the population (Meyer et al., 2011; Jurmain et al., 2012). Indeed, individuals vary significantly in biomechanical properties, even when subsistence activities are expected to be highly characterizing (see below), and relatively homogenous within sexes, as among hunter-gatherers (Kelly, 2000, 2013). It would be therefore not advisable to generalize on Mesolithic activity patterns in Liguria based on Nasino’s results. However, the pattern of robusticity shown by Nasino, when contrasted with other prehistoric foragers, can shed light on the osteobiography of this individual.

Middle to Late Upper Paleolithic hunter-gatherers show similar activity-related adaptations at a European level. Generally, the upper limb shows exceptionally high levels of humeral asymmetry in biomechanical rigidity, especially in males, to the point that they resemble athletes specialized in unimanual sports (e.g. throwing or tennis): this has been attributed to a hunting technology based on throwing weapons (Churchill et al., 1996; Sparacello et al., 2017). The lower limbs appear to be adapted to high levels of terrestrial mobility (Holt, 1999, 2003; Shaw et al., 2013; Sparacello et al., 2018). In the Mesolithic, upper limb asymmetry decreases dramatically in various European sites (Sladek et al., 2016; Sparacello et al., 2020), possibly due to the widespread abandonment of the throwing technology in favor of the bow as a hunting tool (Sparacello et al., 2020; Sladek et al., 2022). Conversely, mobility patterns of Mesolithic people – as reconstructed from biomechanical properties – were more varied, with a strong signal of decreased mobility shown in those areas where coastal resources were exploited (Holt, 1999, 2003), and the retention of characteristics similar to Paleolithic foragers where the focus was on terrestrial resources (Sparacello et al., 2018, 2020).

Albeit Nasino 2 body proportions are normal for a Mesolithic female, size-adjusted mechanical properties of her long bones – evaluated via subperiosteal CSG – are characterized by extreme gracility, to the point that it would be more appropriate to discuss the results in terms of paleopathology rather than adaptations to activity patterns. Furthermore, in a context of extreme gracility, shape indices (Holt, 1999, 2003; Shaw and Stock, 2009a, Sparacello et al., 2018) and inter-limb comparisons (Shaw and Stock 2009b; Shaw and Stock, 2013; Sparacello et al., 2017) do not provide relevant information on activity. For example, Nasino’s humeral residual strength is significantly below the range of variation of the comparative sample. The humeral asymmetry shown by Nasino – the highest in the Mesolithic sample, albeit not particularly high – cannot therefore be attributed to strenuous unimanual activities. A similar conclusion can be drawn for the lower limb data, which show low robusticity, especially in the tibia, and contrasting shape indices: low in the femur (Ix/Iy below 1, i.e. the lowest value in the comparative sample), and high in the tibia (comparable to the ones shown by MUP and LUP males). While low femoral shape index is compatible with low mobility levels (Holt, 1999, 2003; Marchi et al., 2006, 2011; Sládek et al., 2006; Sparacello et al., 2011), a highly platycnemic tibia like the one shown by Nasino is often associated to high mobility patterns (Ruff and Hayes, 1983; Sládek et al., 2006b; Shaw and Stock 2009a), but it has been shown that the shape of the tibia must be interpreted together with tibial robusticity to make relevant inferences (Shaw and Stock 2009a; Sparacello et al., 2018).

The gracility in subperiosteal properties of Nasino 2 suggests a lack of strenuous activities, and its patterning provides insights on the osteobiography of this Mesolithic female. In fact, the humerus is the most gracile bone, followed by the tibia and the humerus. Humeral robusticity was probably directly influenced by the trauma suffered by the right wrist, which most likely limited the mobility of the forearm and hand, as discussed in the main text of this study. Nasino 2 probably could not perform a series of tasks that would have increased her upper limb robusticity. However, lower limb robusticity should have not been influenced by the trauma, but by terrestrial mobility and terrain properties (Carlson and Marchi, 2014). One possibility would be that Nasino 2 was a sedentary individual, yet the isotopic data suggest that her diet was based on resources acquired at high elevation, distant from the area she was buried.

Another, more likely, possibility is that the pattern of gracility shown by Nasino 2 is due to a prolonged period of severely limited activity during adolescence, possibly around the time she suffered the trauma to the forearm, leading to a failure to attain her full growth potential in cross-sectional diaphyseal size. It has been shown that most subperiosteal growth takes place during the pre- and peri-pubertal periods (Frisancho et al., 1970; Garn, 1970), and is determined by body size and activity-related mechanical loadings (Lazenby,1990; van der Muelen, 1997; Pearson and Lieberman, 2004; Sparacello and Pearson, 2010). Physical activity during adolescence is therefore one of the main determinants of diaphyseal structural properties scaled by body size (Ruff et al., 1994, 2006b; Bass al., 1998; Haapasalo, 1998; Haapasalo et al., 2000; Pearson and Lieberman, 2004). Metabolic insults such as malnutrition and long-term illness significantly alter bone development by slowing down subperiosteal apposition (Garn et al.,1964, 1969; Himes et al., 1975; Sparacello et al., 2017). Clinical data and animal models show that, during development, reduced mechanical loadings (van der Meulen et al., 1995; Morey-Holton and Globus, 1998), compromised motor functions (Wren et al., 2011), or low levels of activity due to disease (Kovacs, 2008) result in smaller long bone cross sections. In addition, it appears that long-term conditions result in smaller total areas, with minor effects on the medullary size (Himes et al., 1975; Glick et al., 1981; Bozzini et al., 2013), while more severe insults lead to increased total area and especially medullary area, leading to thinner cortices (Garn et al., 1969; Di Vasta et al., 2007). Smaller cross sections and normal body dimensions in Nasino 2 would be explained by the fact that diaphyseal cross-sectional size appears to be more sensitive to environmental factors, and less genetically canalized, than bone length and articular size (Ruff and Runestad, 1992; Ruff et al., 1993; Auerbach and Ruff, 2006; but see Lieberman et al.,2001). Finally, the pattern of gracility shown in the lower limb – with the femur not particularly gracile and the tibia showing the lowest mechanical strength – is compatible with the observation that differences in modeling responses can occur across ontogeny for the two distal segments (Pearson et al., 2014), with the tibia taking longer to reach adult values (Sparacello et al., 2010), possibly due to greater constrains for tissue economy in the distal segment (Stock, 2006). The marked discrepancy in the cross-sectional shape of the femur and the tibia, albeit common (Pearson at al., 2014), would also be compatible with the fact that the age-related drift pattern from a semi-circular shape to a more antero-posterior oriented shape appears earlier during the ontogeny of the tibia compared to that of the femur (Gosman et al., 2013).

However, these results do not necessarily suggest that Nasino 2 was inactive at the time of death: the effect of increased mechanical loading from mid-adolescence through early adulthood has an effect mainly on the endosteal surface, leading to a contraction of the medullary cavity (Ruff et al., 1994). Conversely, prolonged inactivity and immobilization after the completion of development leads to thinner cortical bone due to medullary area expansion, with little or no change in total area (e.g. Modlesky et al., 2005). Therefore, activity after the trauma would lead to changes in cross-sections that would not be detectable using the Solid CSG method. The radiographic examination of the right forearm does not indicate cortical thinning (Figure 5 in the main text and in Supplementary Information1). The breakage of the left humerus around midshaft allowed for an evaluation of the cortical thickness of this skeletal element, which was compared with a sample of Middle and Late Upper Paleolithic Europeans (Figure S8). Nasino 2 shows a relative cortical thickness that is among the highest in the sample, suggesting medullary stenosis. This is compatible with a vigorous use of the upper limb during adulthood, after the period of halted periosteal apposition during adolescence that was previously hypothesized.


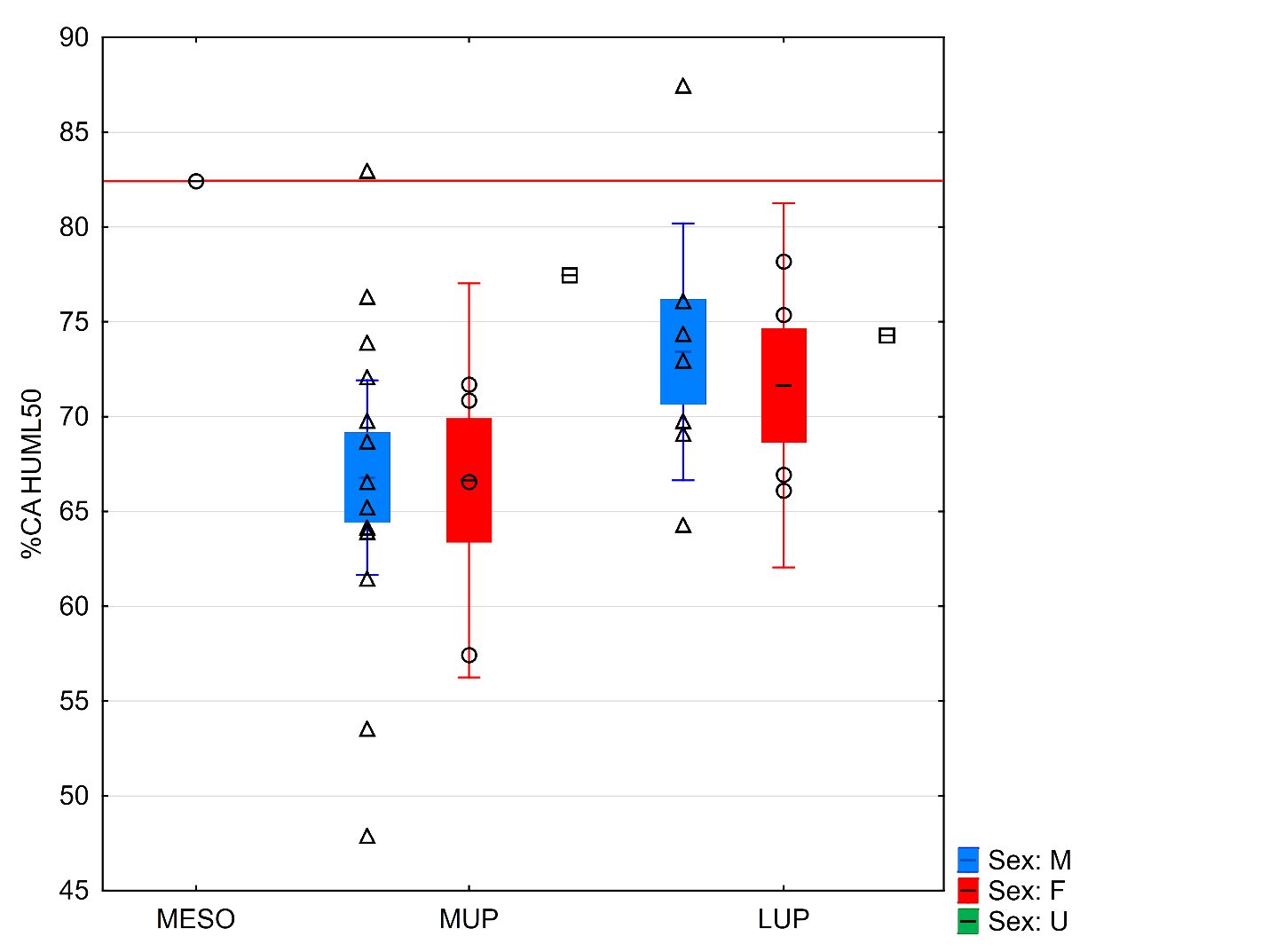


Figure S8 – Cross-sectional percent cortical area of the left humerus (%CA) of Nasino 2 (NAS) at midshaft, compared with a sample of European Middle Upper Paleolithic (MUP; n=19) and Late Upper Paleolithic (LUP; n=12). The box/whisker indicates the mean, the standard error, and the 95% confidence interval.

**Literature Cited**

Auerbach RM, Ruff CB. 2006. Limb bone bilateral asymmetry: variability and commonality among modern humans. J Hum Evol 50:203-218.

Bass SG, Pearce M, Hendrich E, Delmas PD, Harding A, Seeman E. 1998. Exercise before puberty may confer residual benefits in bone density in adulthood: studies in active prepubertal and retired female gymnasts. J Bone Miner Res 13:500-7.

Bozzini C, Picasso EO, Champin GM, Alippi RM, Bozzini CE. 2013.Mechanical testing at whole-bone level of the femur in immature rats stunted by cornstarch consumption. Food Funct 4:1453.

Carlson K, Marchi D (eds). 2014. Reconstructing mobility: environmental, behavioral, and morphological determinants. New York: Springer.

Churchill SE. 1994. Human upper body evolution in Eurasian later Pleistocene, PhD Dissertation, University of New Mexico, Albuquerque.

Churchill SE, Weaver AH, Niewoehner WA. 1996. Late Pleistocene human technological and subsistence behavior. Quatern Nova 6:413-447.

Di Vasta AD, Beck TJ, Petit MA, Feldman HA, Le Boff MS, Gordon CM. 2007. Bone cross-sectional geometry in adolescents and young women with anorexia nervosa: a hip structural analysis study. Osteoporos Int 18:797-804.

Formicola V, Franceschi M. 1996. Regression equations for estimating stature from long bones of early Holocene European samples. Am J Phys Anthropol 100:83-88. [https://doi.org/10.1002/(SICI)1096-8644(199605)100:1<83::AIDAJPA8>3.0.CO;2-E](https://doi.org/10.1002/(SICI)1096-8644(199605)100:1%3c83::AIDAJPA8%3e3.0.CO;2-E)

Formicola V, Giannecchini M. 1999. Evolutionary trends of stature in Upper Palaeolithic and Mesolithic Europe. J Hum Evol 36: 319-333. <https://doi.org/10.1006/jhev.1998.0270>

Frisancho RA, Garn SM, Ascoli W. 1970. Subperiosteal and endosteal bone apposition during adolescence. Hum Biol 42:639-664.

Garn SM, Rohmann CB, Behar M, Viteri F, Guzman MA. 1964. Compact bone deficiency in protein-calorie malnutrition. Science 145:1444-1445.

Garn SM, Guzman MA, Wagner B. 1969. Subperiosteal gain and endosteal loss in protein-calorie malnutrition. Am J Phys Anthropol 30:153.155.

Garn SM. 1970. The Earlier Gain and the Later Loss of Cortical Bone. Springfield: Charles C Thomas.

Glick PL, Rowe DJ. 1981. Effects of chronic protein deficiency on skeletal development of young rat.s Calcif Tissue Int 33:223-231.

Gosman JH, Hubbell ZR, Shaw CN, Ryan TM. 2013. Development of Cortical Bone Geometry in the Human Femoral and Tibial Diaphysis. Anat Rec 296:774-787. https://doi.org/10.1002/ar.22688

Haapasalo H. 1998. Physical activity and growing bone: development of peak bone mass with special reference to the effects of unilateral activity. Ann Chir Gynaecol 87:250-252.

Haapasalo H, Kontulainen S, Sievanen H, Kannus P, Jarvinen M, Vuori I. 2000. Exercise-induced bone gain is due to enlargement in bone size without a change in volumetric bone density: a peripheral quantitative computed tomography study of the upper arms of male tennis players. Bone 27:351-357.

Himes JH, Martorell R, Habicht JP, Yarbrough C, Malina RM, Klein RE. 1975. Patterns of cortical bone growth in moderately malnourished preschool children. Hum Biol 47:337-350.

Holliday TW. 1995. Body size and proportions in the Late Pleistocene western Old World and the origins of modern humans, PhD Dissertation, University of New Mexico, Albuquerque.

Holliday TW. 1997. Body proportions in Late Pleistocene Europe and modern human origins. J Hum Evol 32:423–447. https://doi.org/10.1006/jhev.1996.0111

Holliday TW. 2002. Body size and postcranial robusticity of European Upper Paleolithic hominins. J Hum Evol 43:513-528. <https://doi.org/10.1006/jhev.2002.0590>

Holt BM. 1999. Mobility in Upper Paleolithic and Mesolithic Europe: biomechanical evidence from the lower limb. Dissertation, University of Missouri.

Holt BM. 2003. Mobility in Upper Paleolithic and Mesolithic Europe: evidence from the lower limb. Am J Phys Anthropol 122:200-15.

Jurmain R, Alves Cardoso F, Henderson C, Villotte S. 2012. Bioarchaeology’s holy grail: the reconstruction of activity. In: Grauer AL, editor. A companion to paleopathology. New York: Wiley-Blackell. Pp. 531–552.

Kelly RL. 2000. Elements of behavioral ecological paradigm for the study of prehistoric hunter-gatherers. In: Schiffer MB, editor. Social Theory in Archaeology. Salt Lake City: The University of Utah Press. Pp. 63-78.

Kelly RL. 2013. The Lifeways of hunter-gatherers. The foraging spectrum. Cambridge: Cambridge University Press.

Kovacs CS. 2008. Hemophilia, low bone mass, and osteopenia/osteoporosis. Transfus Apher Sci 38:33-40.

Lazenby RA. 1990. Continuing periosteal apposition II: the significance of peak bone mass, strain equilibrium, and age related activity differentials for mechanical compensation in human tubular bones. Am J Phys Anthropol 82:473–484.

Lieberman DE, Devlin MJ, Pearson OM. 2001. Articular area response to mechanical loading: effects of exercise, age, and skeletal location. Am J Phys Anthropol 116:266-277.

Macintosh AA, Davies TG, Ryan TM, Shaw CN, Stock JT. 2013. Periosteal versus true cross-sectional geometry: a comparison along humeral, femoral, and tibial diaphysis. Am J Phys Anthropol 150:442-452.

Macintosh AA, Stock JT. 2019. Intensive terrestrial or marine locomotor strategies are associated with inter- and intra-limb bone functional adaptation in living female athletes. Am J Phys Anthropol 168:566-581.

Marchi D, Sparacello VS, Holt BM, Formicola V. 2006. Biomechanical approach to the reconstruction of activity patterns in Neolithic Western Liguria, Italy. Am J Phys Anthropol 131:447-455.

Marchi D, Sparacello VS, Shaw CN. 2011. Mobility and lower limb robusticity of a pastoralist Neolithic population from North-Western Italy. In: Pinhasi R, Stock JY, editors. Human bioarchaeology of the transition to agriculture. New York: John Wiley & Sons. Pp 317-346.

Martin RB, Atkinson PJ. 1977. Age and sex-related changes in the structure and strength of the human femoral shaft. J Biomech 10:223-231.

Meyer C, Nicklisch N, Held P, Fritsch B, Alt KW. 2011. Tracing patterns of activity in the human skeleton: An overview of methods, problems, and limits of interpretation. J Comp Hum Biol 62:202-217.

Morey-Holton ER, Globus RK. 1998. Hindlimb unloading of growing rats: a model for predicting skeletal changes during space flight. Bone 22(5):83-88.

Nagurka ML, Hayes WC. 1980. An interactive graphics package for calculating cross-sectional properties of complex shapes. J Biomech 13:59-64.

Pearson OM. 1997. Postcranial morphology and the origin of modern humans. PhD Dissertation, State University of New York at Stony Brook, New York.

Pearson OM, Lieberman DE. 2004. The aging of Wolff’s ‘Law’: ontogeny and response to mechanical loading in cortical bone. Am J Phys Anthropol 47:63-99.

Pearson OM, Petersen TR, Sparacello VS, Daneshvari S, Grine FE. 2014. Activity, “body shape”, and cross-sectional geometry of the femur and tibia. In: Carlson K, Marchi D, editors. Reconstructing mobility: environmental, behavioral, and morphological determinants. New York: Springer. doi.org/10.1007/978-1-4899-7460-0_8. Pp. 133-153.

Pinhasi R, Stock J, editors. 2011. Human bioarchaeology of the transition to agriculture. New York: Wiley-Liss.

Rhodes JA, Knüsel CJ. 2005. Activity-related skeletal change in medieval humeri: cross-sectional and architectural alterations. Am J Phys Anthropol 128:536-46.

Ruff CB. 2000. Body size, body shape, and long bone strength in modern humans. J Hum Evol 38:269-290.

Ruff CB. 2002. Long bone articular and diaphyseal structure in Old World monkeys and apes. I: locomotor effects. Am J Phys Anthropol 119:305-42

Ruff CB. 2018. Quantifying skeletal robusticity. In: Ruff CB, editor. Skeletal variation and adaptation in Europeans: Upper Paleolithic to the Twentieth Century. New York: John Wiley & Sons. Pp. 39-47.

Ruff C, Hayes W. 1988. Sex differences in age-related remodeling of the femur and tibia. J Orthop Res 6:886-896.

Ruff CB, Holt BM, Sládek V, Berner M, Murphy W, zur Nedden D, Seidler H, Recheis W. 2006a. Body size, body proportions, and mobility in the Tyrolean ‘Iceman’. J Hum Evol 51:91-101.

Ruff CB, Holt B, Trinkaus E. 2006b. Who’s afraid of the big bad Wolff? ‘Wolff’s law’ and bone functional adaptation. Am J Phys Anthropol 129:484-498. <https://doi.org/10.1002/ajpa.20371>

Ruff CB, Runestad J. 1992. Primate limb bone structural adaptations. Ann Rev Anthropol 21: 407-433.

Ruff CB, Trinkaus E, Walker A, Larsen CS. 1993. Postcranial robusticity in Homo I: temporal trends and mechanical interpretation. Am J Phys Anthropol 91:21-54.

Ruff CB, Walker A, Trinkaus E. 1994. Postcranial robusticity in Homo, III: ontogeny. Am J Phys Anthropol 93:35-54.

Scarsini C, Messeri P. 1974. Lo scheletro n. 2 di età neolitica, rinvenuto all’”Arma di Nasino” in Val Pennavaira (Savona). Riv Sci Preist 29(1):153-178.

Shaw C, Stock J. 2009a. Intensity, repetitiveness, and directionality of habitual adolescent mobility patterns influence the tibial diaphysis morphology of athletes. Am J Phys Anthropol 140:149-159.

Shaw C, Stock J. 2009b. Habitual throwing and swimming correspond with upper limb diaphyseal strength and shape in modern human athletes. Am J Phys Anthropol 140:160-172.

Shaw CN, Stock JT. 2013. Extreme mobility in the Late Pleistocene? Comparing limb biomechanics among fossil Homo, varsity athletes and Holocene foragers. J Hum Evol 64:242-249.

Sládek V, Berner M, Sailer R. 2006a. Mobility in Central European Late Eneolithic and Early Bronze Age: femoral cross-sectional geometry. Am J Phys Anthropol 130:320-332.

Sládek V, Berner M, Sailer R. 2006b. Mobility in Central European Late Eneolithic and Early Bronze Age: tibial cross-sectional geometry. J Archaeol Sci 33:470-482.

Sládek V, Ruff CB, Berner M, Holt B, Niskanen V, Schuplerová E, Hora M. 2016. The impact of subsistence changes on humeral bilateral asymmetry in Terminal Pleistocene and Holocene Europe. J Hum Evol 92:37-49.

Sládek V, Hora M, Véle D, Rocek T. 2022. Bow and muscles: observed muscle activity in archers and potential implications for habitual activity reconstruction. J Archaeol Sci 144:105638.

Sparacello VS, d’Ercole V, Coppa A. 2015. A bioarchaeological approach to the reconstruction of changes in military organization among Iron Age Samnites (Vestini) from Abruzzo, central Italy. Am J Phys Anthropol 156:305-16.

Sparacello VS, Pearson OM. 2010. The importance of accounting for the area of the medullary cavity in cross-sectional geometry: a test based on the femoral midshaft. Am J Phys Anthropol 143:612-624.

Sparacello VS, Pearson OM, Coppa A, Marchi D. 2011. Changes in robusticity in an Iron Age agropastoral group: the Samnites from the Alfedena necropolis (Abruzzo, Central Italy). Am J Phys Anthropol 144:119-130.

Sparacello VS, Pearson OM, Cowgill LW. 2010. Growing up in the Gravettian: ontogeny of cross-sectional geometry in the lower limb. Am J Phys Anthropol [Suppl] 50:169.

Sparacello VS, Samsel M, Villotte S, Varalli A, Schimmenti V, Sineo L. 2020. Inferences on Sicilian Mesolithic subsistence patterns from cross-sectional geometry and entheseal changes. Archaeol Anthropol Sci 12(5):101.

Sparacello VS, Villotte S, Shackelford LL, Trinkaus E. 2017. Patterns of Humeral Asymmetry among Late Pleistocene Humans. C R Palevol 16(5-6):680-689.

Sparacello VS, Villotte S, Shaw CN, Fontana F, Mottes E, Starnini E, Dalmeri G, Marchi D. 2018. Changing mobility patterns at the Pleistocene-Holocene transition: the biomechanics of the lower limb of Italian Gravettian and Mesolithic individuals. In: Cristiani E, Borgia V, editors. Paleolithic Italy: Advanced studies on early human adaptations in the Apennine Peninsula. Leiden, Sidestone Press. Pp. 357-396.

Stock JT. 2006. Hunter-gatherer postcranial robusticity relative to patterns of mobility, climatic adaptation, and selection for tissue economy. Am J Phys Anthropol 131:194-204.

Stock JT, Shaw CN. 2007. Which measures of skeletal robusticity are robust? A comparison of external methods of quantifying diaphyseal strength to cross-sectional geometric properties. Am J Phys Anthropol 134:412-423.

Trinkaus E, Ruff CB. 2012. Femoral and tibial diaphyseal cross-sectional geometry in Pleistocene Homo. PaleoAnthropology 2012:13-62.

van der Muelen MCH. 1997. Diaphyseal bone growth and adaptation: models and data. In: Lowet G, Rüegsegger P, Weinans H, Meunier A, editors. Bone Research in Biomechanics. Clifton: IOS Press. Pp. 17-23.

Varalli A, Villotte S, Dori I, Sparacello VS. 2020. New insights into activity-related functional adaptations and entheseal changes in Neolithic Liguria (northwestern Italy). Bull Mem Soc Anthropol Paris 32:34–58. 10.3166/bmsap-2020-0072.

Villotte S, Samsel M, Sparacello VS. 2017. The paleobiology of the two adult skeletons from Baousso da Torre (Bausu da Ture) (Liguria, Italy): implications for our understanding of Gravettian lifestyle. Comptes Rendus Palevol 16:462-473.

Wren TAL, Lee DC, Kay RM, Dorey FJ, Gilsanz V. 2011. Bone density and size in ambulatory children with cerebral palsy. Dev Med Child Neurol 53:137-141.
